# Global and seasonal variation of marine phosphonate metabolism

**DOI:** 10.1038/s41396-022-01266-z

**Published:** 2022-06-23

**Authors:** Scott Lockwood, Chris Greening, Federico Baltar, Sergio E. Morales

**Affiliations:** 1 Department of Microbiology and Immunology, University of Otago, PO Box 56, Dunedin, 9054 New Zealand; 2 Department of Marine Science, University of Otago, PO Box 56, Dunedin, 9054 New Zealand; 3 Department of Microbiology, Biomedicine Discovery Institute, Monash University, Clayton, VIC 3800 Australia; 4 Department of Functional and Evolutionary Ecology, University of Vienna, Vienna, Austria

**Keywords:** Water microbiology, Microbial ecology, Microbial biooceanography, Metagenomics

## Abstract

Marine microbial communities rely on dissolved organic phosphorus (DOP) remineralisation to meet phosphorus (P) requirements. We extensively surveyed the genomic and metagenomic distribution of genes directing phosphonate biosynthesis, substrate-specific catabolism of 2-aminoethylphosphonate (2-AEP, the most abundant phosphonate in the marine environment), and broad-specificity catabolism of phosphonates by the C-P lyase (including methylphosphonate, a major source of methane). We developed comprehensive enzyme databases by curating publicly available sequences and then screened metagenomes from TARA Oceans and Munida Microbial Observatory Time Series (MOTS) to assess spatial and seasonal variation in phosphonate metabolism pathways. Phosphonate cycling genes were encoded in diverse gene clusters by 35 marine bacterial and archaeal classes. More than 65% of marine phosphonate cycling genes mapped to Proteobacteria with production demonstrating wider taxonomic diversity than catabolism. Hydrolysis of 2-AEP was the dominant phosphonate catabolism strategy, enabling microbes to assimilate carbon and nitrogen alongside P. Genes for broad-specificity catabolism by the C-P lyase were far less widespread, though enriched in the extremely P-deplete environment of the Mediterranean Sea. Phosphonate cycling genes were abundant in marine metagenomes, particularly from the mesopelagic zone and winter sampling dates. Disparity between prevalence of substrate-specific and broad-specificity catabolism may be due to higher resource expenditure from the cell to build and retain the C-P lyase. This study is the most comprehensive metagenomic survey of marine microbial phosphonate cycling to date and provides curated databases for 14 genes involved in phosphonate cycling.

## Introduction

Phosphorus (P) is an essential nutrient for all organisms required for the synthesis of nucleic acids, ATP, phospholipids, and numerous metabolites. In turn, P-availability is a major factor governing oceanic productivity [1–4]. Dissolved inorganic phosphate (DIP) is regarded as the most bioavailable form of P, yet DIP is often limited in marine surface waters [5, 6]. Bacterioplankton adapt to DIP scarcity by scavenging P from a variety of organic compounds [7], collectively labelled dissolved organic phosphorus (DOP). Oceanic DOP concentrations are often much higher than DIP [3, 8] and can influence microbial community growth and composition [9, 10], primary productivity, nitrogen fixation, and CO_2_ uptake [11, 12].

Phosphonates, characterized by the chemically inert C-P bond, make up to 25% of the DOP pool [13, 14]. The stability of the C-P bond provides resistance to enzymatic and abiotic degradation processes. Phosphonates serve many functions, from incorporation into lipids, glycans, and glycoproteins to increase structural strength and protect against enzymatic degradation, to acting as antimetabolites for structurally analogous phosphate esters or carboxylic acids [15, 16]. Microbes with genes for phosphonate catabolism can utilize phosphonates as P sources and, in certain instances, carbon and nitrogen sources [17, 18]. Phosphonate catabolism requires cleavage of the C-P bond, which oxidizes phosphonates (+3 oxidative state) to inorganic phosphate (Pi) or phosphite ions (both + 5 oxidative state), and plays a central role in the oceanic P redox cycling [19, 20].

**Fig. 1:**
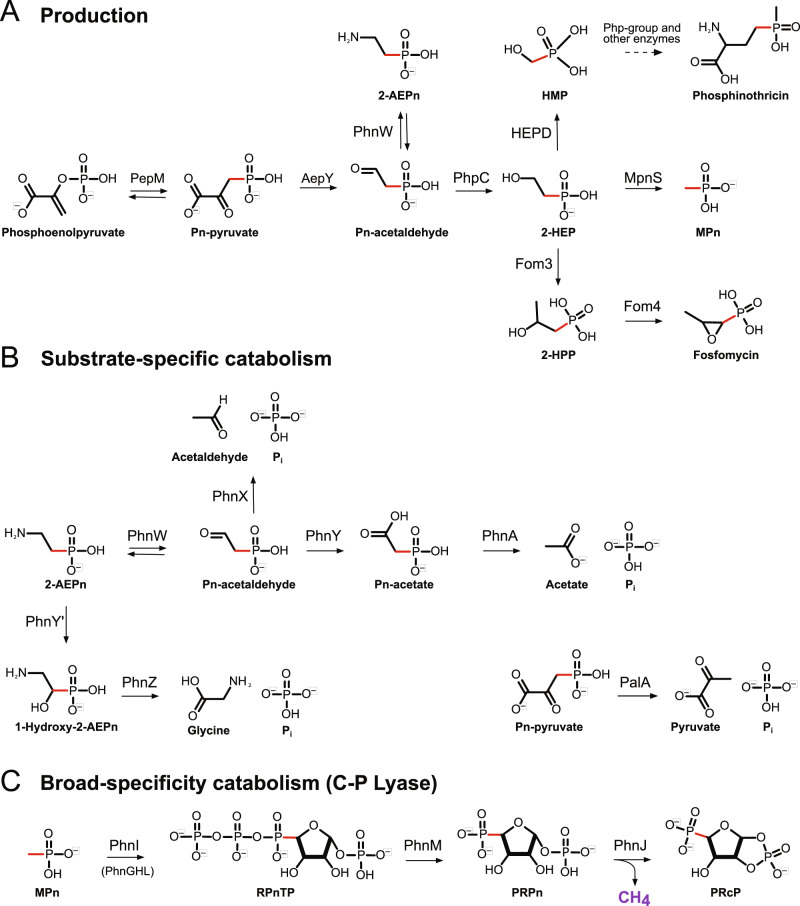
Enzymatic pathways for phosphonate production and degradation. Biological transformation pathways for phosphonate **A** production, **B** substrate-specific catabolism, and **C** broad-specificity catabolism. Enzyme abbreviations within grey boxes, substrate abbreviations under molecular structure, and C-P bond is highlighted in red. MPn used as example substrate for broad-specificity catabolism by C-P lyase to demonstrate production of methane. Enzyme abbreviations as follows: phosphoenolpyruvate mutase (*pepM*), phosphonopyruvate decarboxylase (*aepY*), phosphonoacetaldehyde reductase (*phpC*), 2-hydroxyethylphosphonate dioxygenase (*hepD*), methylphosphonate synthase (*mpnS*), phosphonoacetaldehyde methylase (*fom3*), (S)-2-hydroxypropylphosphonic acid epoxidase (*fom4*), 2-AEP—pyruvate transaminase (*phnW*), phosphonoacetaldehyde hydrolase (*phnX*), phosphonoacetaldehyde dehydrogenase (*phnY*), phosphonoacetate hydrolase (*phnA*), 2-aminoethylphosphonate dioxygenase (*phnY’*), 2-amino-1-hydroxyethylphosphonate dioxygenase (*phnZ*), phosphonopyruvate hydrolase (*palA*), C-P lyase core-complex subunit I (*phnI*), C-P lyase core-complex subunit J (*phnJ*), Alpha-D-ribose 1-methylphosphonate 5-trisphosphate diphosphatase (*phnM*). Substrate abbreviations as follows: phosphoenolpyruvate (PEP), phosphonopyruvate (Pn-pyruvate), phosphonoacetaldehyde (Pn-acetaldehyde), phosphonoacetate (Pn-acetate), 2-aminoethylphosphonate (2-AEP), 2-hydroxyethylphosphonate (2-HEP), hydroxymethylphosphonic acid (HMP), methylphosphonate (MPn), 2-hydroxypropylphosphonic acid (2-HPP), alpha-D-ribose 1-methylphosphonate 5-triphosphate (RPnTP), alpha-D-ribose-1-phosphonate-5-phosphate (PRPn), alpha-D-ribose-1,2-cyclic-phosphate-5-phosphate (PRcP).

Despite the great variety in phosphonates and phosphonate-conjugated compounds, there are two phosphonates of particular relevance and interest in the marine environment: 2-aminoethylphosphonate (2-AEP) and methylphosphonate (MPn) (Fig. 1A). 2-AEP is the most universally abundant naturally occurring phosphonate [21], particularly in the marine environments given it is conjugated to glycans [22], lipids [23], and proteins [24] of many marine organisms. Microbes can utilize 2-AEP as their only source of P and, if necessary, carbon and nitrogen [25, 26]. Mineralisation of 2-AEP has been recently shown to be an essential step in the marine P redox cycle [27]. MPn is a key marine phosphonate and, together with its precursor 2-hydroxyethylphosphonate (2-HEP), accounts for up to 20% of P associated with high molecular weight dissolved organic matter (HMWDOM) attached to polysaccharides as esters [28]. The function of MPn in the cell is not explicitly known, though previous work suggests that microbes may synthesize exopolysaccharides modified with MPn [29]. Importantly, MPn catabolism results in aerobic production of methane [30]; this gas is released at sufficient levels to influence the atmospheric methane cycle, thereby explaining the “oceanic methane paradox” [31, 32] (Fig. 1C).

The biosynthesis of all phosphonates requires a common C-P bond-forming reaction catalysed by phosphoenolpyruvate mutase [33] (PepM) (Fig. 1A). The conversion of phosphoenolpyruvate to phosphonopyruvate (Fig. 1A) is thermodynamically unfavourable reaction, so many phosphonate-producing microbes couple forming the C-P bond with an irreversible conversion to phosphonoacetaldehyde [33]. Several possible biosynthesis pathways can act upon phosphonoacetaldehyde, leading to the production of many different phosphonates including phosphinothricin (herbicide), fosfomycin (antibiotic), or decorations for protein exports (Fig. 1A).

Microbes catabolize phosphonates through a range of strategies, including substrate-specific catabolism through specialized hydrolases/dioxygenases and broad-specificity catabolism by the C-P lyase that can hydrolyse a broad range of phosphonates (Fig. 1B). The four prevalent phosphonohydrolases are PalA [34], PhnA [35], PhnX [36], and PhnZ [37, 38], the latter three targeting 2-AEP (Fig. 1B). PhnA and PhnX require PhnW as the initial step in catabolism [39] to transform 2-AEP into phosphonoacetaldehyde, and PhnA requires an intermediate step carried out by PhnY [40] (Fig. 1B). PhnZ does not require PhnW but rather uses an Fe(II)-dependent dioxygenase (PhnY’) as an intermediate step for 2-AEP catabolism (Fig. 1B). Expression of phosphonohydrolases can be Pi-insensitive and likely substrate-inducible mediated by LysR-type transcriptional regulators [26, 41, 42], reflecting the ability of some microbes to use 2-AEP not only as a sole source of P, but also C and N [17, 43]. The versatile C-P lyase [43–47] facilitates microbial assimilation of P from a wide-range of phosphonate substrates, including MPn (Fig. 1C). The lyase is synthesized from a 14 gene operon (PhnGHIJKLMNOP) that is induced under phosphate starvation through the *pho* regulon [48, 49]. Phosphonate activation and degradation occurs through a series of steps as detailed in Fig. 1C for MPn [50, 51].

Marine microbial production and utilization of phosphonates is well-documented. However, previous studies either focused on a subset of genes or overlooked phosphonate production, leading to fragmented and incomplete perspectives of marine microbial phosphonate cycling [26, 37, 41, 45, 52–57]. In addition, the seasonal and spatial variations of this metabolism have not been thoroughly investigated. In this study, we addressed these knowledge gaps by (i) characterizing global spatial trends for phosphonate cycling potential (gene abundance) in the surface and deep ocean, (ii) identifying taxa involved in the different phosphonate production and consumption pathways, and (iii) determining the effect of seasonality on the potential for phosphonate cycling activity. To achieve this, we curated databases from the publicly available JGI IMG/MER online database and the Global Ocean Reference Genome Tropics (GORG-Tropics) database for genes involved in phosphonate production (*pepM*, *aepY, phpC, mpnS, hepD*) alongside substrate-specific catabolism (*palA*, *phnA*, *phnW*, *phnX*, *phnY, phnZ*) and broad-specificity catabolism (*phnI*, *phnJ, phnM*). Databases were screened against metagenomes and associated metatranscriptomes from the publicly available TARA Oceans Project to elucidate global trends in gene abundance and taxonomy, and the local Munida Time-Series Transect (MOTS) to investigate spatiotemporal variation. MOTS is a transect crossing the Southland Front, sampling contrasting water masses across a 65 km distance offshore [58, 59] (Supplementary Fig. 1). Our findings confirm the global prevalence of phosphonate production and catabolism in marine microbial communities, and demonstrates its higher prevalence during winter months (than in summer) and in mesopelagic waters (than in epipelagic waters).

## Methods

### Database construction

We downloaded amino acid sequences of all putative phosphonate cycling enzymes (PepM, AepY, PhpC, MpnS, HepD, PalA, PhnA, PhnW, PhnX, PhnY, PhnZ, PhnI, PhnJ, PhnM) from the Joint Genome Institute (JGI) Integrated Microbial Genomes and Microbiomes (IMG/MER) database [60] and the Global Ocean Reference Genomes Tropics (GORG-Tropics) database [61]. Preliminary screening for genes using reliable annotations (KEGG, TIGRfam, and/or COG) (Supplementary Table S1) revealed 9,454 genomes from the IMG/MER database with genes for phosphonate cycling. Restricting the gene search to the set of 9454 genomes, protein sequences were retrieved by aforementioned annotation during March 2021. Utilizing the same annotations, we recovered protein sequences for phosphonate cycling genes from 883 single amplified genomes (SAGs) in the GORG-Tropics database.

For all bioinformatics tools, default settings were used unless otherwise indicated. Following sequence collection, databases were aligned using MUSCLE v3.8.1551 [62]. We verified or removed sequences based on presence/absence of conserved residues identified as essential for catalytic action, substrate binding, or cofactor binding from protein crystallization studies [36, 40, 46, 48, 51, 63–80] (Supplementary Table S2). We curated the databases using the “Sort sequences by character in selected column” function in AliView v1.26 [81] to isolate important residues for comparison. We used the genome neighbourhood browser in the IMG/JGI for verification by synteny for *phpC* and *phnY*, which lacked consistent identification by annotation as both are related to common protein families (Class IV Fe-alcohol dehydrogenases and aldehyde oxidoreductases, respectively). The presence of other phosphonate cycling genes in close proximity was required for the addition of any *phpC* and *phnY* sequences to the databases.

Gene taxonomy and NCBI taxon ID were retrieved from the IMG/MER genome database and GORG-Tropics catalogue. Additional metadata for IMG/MER genomes was collected spanning: Ecosystem, Ecosystem Category, Ecosystem Type, Habitat, Energy Source, Metabolism, Motility, and Oxygen Requirement (Supplemental Dataset S1) to identify any marine genomes from the IMG/MER collection, though habitat and ecosystem metadata was only available for 45.5% (4305) of the IMG/MER genomes. Following curation based on conserved residues, we re-aligned the database fasta files with MUSCLE v3.8.1551 and trimmed sequences using trimAl v1.4 [82] with the automated -gappyout setting to generate redundant databases. We then created non-redundant (nr) databases by dereplication to 99% using CD-HIT v4.8.1 [83]. The nr-databases were used for all analysis except investigating gene co-occurrence where we utilized the redundant databases. We constructed phylogenetic trees for each nr-database with FastTree v2.1.10 [84] using the LG + CAT amino acid substitution model over 100 bootstraps and refined the trees using RAxML v8.2.12 [85] with the PROTCATLG model and seed of -p 1994 (Supplementary Figs. S3, S4, S5). Databases can be found within Supplementary Files S1 (nr) and Supplementary Files S2 (redundant) and with metadata for genes and genomes, including a breakdown of phosphonate cycling genes found in each genome, located in Supplementary Dataset S1. Each nr-database’s phylogenetic tree is available in Newick tree format in Supplementary Files S3.

### Retrieval of TARA metagenomes and metatranscriptomes

TARA Oceans Project metagenomes [86] were retrieved and divided into geographic clusters following the categorization detailed in Delmont et al. (Supplementary Fig. 2). Our sample-grouping scheme retains the same twelve location clusters and sample sites within each location. However, the number of metagenomes retrieved is expanded to include 51 mesopelagic (MES) alongside 101 surface (SUR) and 61 deep chlorophyll maximum (DCM) samples. No mesopelagic samples from the TARA survey of Mediterranean Sea samples (MED) were available, restricting analysis to surface and DCM samples. We also retrieved any metatranscriptomes sequenced from samples in our TARA metagenome dataset for comparison of gene abundance versus transcription. Further information on the selection of TARA samples, including INSDC run accession number, Pangaea Sample ID, and environmental metadata, can be found in Supplementary Table S3.

### Munida Time-Series Transect sampling and sequencing

MOTS samples in this study represent winter (June–August) and summer (December–February) periods, capturing the maximum degree of seasonal variability in the system [87, 88]. Sampling dates and metadata can be found in Supplementary Table S4. At each sampling, duplicate 1 L samples of seawater were collected from surface stations representing sub-tropical and sub-Antarctic waters (STW and SAW), with an additional sample collected from 500 m deep in the sub-Antarctic station (SAW-MES). Samples were filtered on to 0.22 µm pore-size MF-Millipore membrane filter by vacuum pumping and immediately frozen and stored at −80 °C. For DNA extractions, filters were cut into small strips with a razor blade and processed using the MoBio Powersoil DNA Isolation Kit. Libraries were prepared with the ThruPLEX DNA-sequencing (DNA-seq) Kit (Takara) and sequenced on three lanes of the HiSeq2500 machine (Illumina). Twenty-one samples were sequenced for a total of 89.9 Gb (2.8 Gb average per sample after quality control transformed to FASTA format). The raw reads were submitted to the NCBI-SRA archive and are available under the BioProject PRJNA605648: Munida Microbial Observatory Time-Series (MOTS) [HiSeq].

### Metagenome analysis

Quality control was performed on metagenomes (TARA and MOTS) and metatranscriptomes (TARA) using tools from the BBMap [89] 38.71 release. Meta-genomes/transcriptomes were initially condensed by grouping overlapping reads into clumps using Clumpify [89]. Next we used BBDuk [89] to perform adapter trimming with options *ktrim* = *r k* = *23 mink* = *11 hdist* = *1 tpe tbo* and targeted removal of PhiX adapters with options *k* = *31 hdist* = *1*. Reads were then processed with BBMap [89] for removal of human contaminants using a masked version of the human genome HG19 [89]. We processed sequences as paired reads during aforementioned quality control and then concatenated the forward and reverse reads for each sample for gene screening.

Protein searches of each individual database against metagenomes were carried out using DIAMOND v2.0.6.144 [90] with the -k 1 option utilizing the nr-databases as reference. To minimize false positives, hits from the DIAMOND screen were filtered based on percentage identity over a given length. The minimum percentage identity varied by gene (50% - *pepM, aepY, phnAWXZI*; 55% - *phnM*; 60% - *phpC, mpnS, hepD, palA, phnJ*; 65% - *phnY*) to achieve a reliable homology cut-off across a minimum query coverage of 32 (TARA – 100 bp reads) or 40 (MOTS – 125 bp reads) amino acids. Every unique instance of a DIAMOND hit was counted regardless if it was from the forward or reverse reads prior to concatenation.

We normalized phosphonate cycling gene hits by copy number and length following the equation (Hit count)*(Copy number normalizing factor)*(Gene length normalizing factor) to account for the potential of genomes containing multiple copies of a gene and ensure BLAST hits from genes of varying lengths on short-read query could be accurately compared without bias towards longer genes. The copy number normalization factor was calculated by the formula (# of genomes)/(# of sequences) for each nr database. The length normalization factor was determined by the formula (Average length of all sequences in study)/(Average length of sequences in a given database). Both normalization factors for each database can be found in Supplemental Dataset S1.

As a proxy for counting the number of individuals within a given community, we averaged the length-normalized abundance of five conserved single-copy genes (*recA, atpD, tufA, gyrB, hsp70*) [91]. Multiple conserved single-copy genes were used for a more robust estimation of community size and length normalization was calculated based on the length of the shortest gene (*recA*) following the formula (length of *recA*)/(length of subject gene) to avoid overestimation of community size due to BLAST bias towards longer genes. Average relative abundance was calculated by dividing the normalized phosphonate gene hit by the normalized average of the conserved single-copy gene counts.

### Statistical analysis

The Shannon index of each gene database was calculated using the *vegan* package v2.5-6 [92] in R. We performed one-way ANOVA to investigate changes in relative gene abundance between specific genes, depths, and/or locations for both the TARA and MOTS datasets. We fitted linear regression models of the log-transformed relative abundance of each gene to the log-transformed Pi concentration for each sample. Pi values had 0.01 added to the value before the log transformation to allow for inclusion of samples with phosphate concentration below the detectable limit. One sample from MOTS, six from the Southern Indian Ocean (IOS) and two from the Northeast Atlantic Ocean (ANE) were excluded from the linear regression analysis due to missing metadata. Separate regression models were created based solely on either surface or mesopelagic samples from both TARA and MOTS metagenomes. We built an NMDS ordination based upon a Bray-Curtis dissimilarity matrix on the relative gene abundance for all 14 genes in each of the MOTS samples followed up by an ANOSIM to test for significance. Code for statistical analysis can be found at https://github.com/S-P-Lockwood/Lockwood-et-al.-2022.

## Results and discussion

### Proteobacteria are major contributors to marine microbial phosphonate cycling

**Fig. 2:**
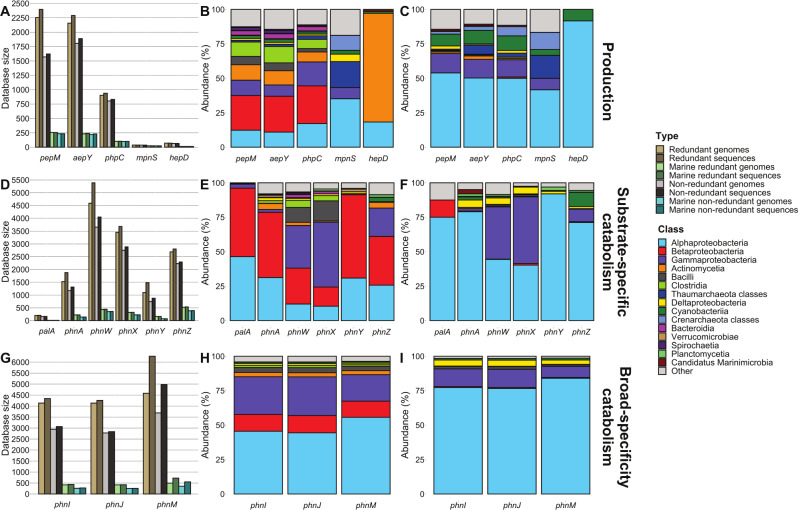
Phosphonate gene and genome count with taxonomic distribution. Number of sequences and genomes collected for study (**A**, **D**, **G**) with distribution of class-level taxa for all redundant sequences (**B**, **E**, **H**) and marine redundant (**C**, **F**, **I**) sequences. Results are shown for selected genes representing phosphonate (**A**–**C**) production, (**D**–**F**) substrate-specific catabolism, and (**G**–**I**) broad-specificity catabolism. The taxa shown are the 15 classes with the highest representation across all databases.

Databases for all putative sequences of genes for phosphonate production (*pepM, aepY, phpC*, *mpnS, hepD*), substrate-specific catabolism (*phnAWXYZ, palA*), and broad-specificity catabolism (*phnIJM*) were created using available public genomes from JGI IMG/MER and GORG-Tropics. Gene identity was verified by the presence of catalytically essential residues (Supplementary Table S2). Phosphonate genes were identified in 10,337 genomes of bacteria and archaea spanning over 100 unique classes, suggesting a wide variety of microorganisms mediate phosphonate production and catabolism (Fig. 2, Supplementary Dataset S1). A high proportion of all collected sequences affiliated with Proteobacteria (Gamma, Alpha, and Beta classes), averaging 52% of the production genes, 78% of substrate-specific catabolism genes, and 88% of broad-specificity catabolism genes before dereplication (Fig. 2).

Of the 10,337 genomes, 1556 (15%) were confirmed to be marine organisms from 35 different classes (Fig. 2, Supplementary Dataset S1). Proteobacteria had even greater representation in the subset of marine genomes, averaging 65% of marine production genes, 88% of marine substrate-specific catabolism genes, and 96% of marine broad-specificity catabolism genes from the redundant databases (Fig. 2). The dominance of Alphaproteobacteria in the marine subset may be attributed to the wide variety of Pelagibacterales bacterium captured in the database, making up 426 (27%) of the 1556 genomes involved in all three categories of phosphonate cycling. Rhodobacterales (*Ascidiaceihabitans* sp., *Roseovarius* sp., *Sulfitobacter* sp., *Labrenzia* sp., and *Phaeobacter* sp.) alongside Rhodospirillales (*Thalassobaculum* sp., *Thalassospira* sp., *Roseospira* sp., *Varunaivibrio* sp., and *Oceanibaculum* sp.) were also highly represented among the marine subset with 214 (14%) and 251 (16%) genomes, respectively (Supplemental Dataset S1), though these taxa primarily show potential for phosphonate catabolism rather than production. Vibrionales were well represented in the JGI IMG/MER marine genome subset with 107 (7%) genomes spanning 59 different species including *Vibrio lentus*, *Vibrio breoganii*, and *Vibrio splendidus*.

### Diverse taxa encode the capacity to produce phosphonate derivatives

Phosphonate production is widespread and distributed throughout many different bacteria and archaea. Genes responsible for the first two steps in phosphonate production, *pepM* and *aepY*, had the broadest taxonomic distribution within the redundant databases (Shannon indices of 2.66 and 2.76) for all genes in this study, distributed with 0.59 and 0.61 evenness from 70 and 72 unique, verified classes, respectively. Their broad distribution further highlights the ubiquity and necessity of phosphonate compounds to microbial life and function across all environments. Within the marine setting, both *pepM* and *aepY* have reference sequences from 22 unique, verified classes which is the second highest class representation in the marine genome subset (Fig. 2). The marine subset of *pepM* and *aepY* also have the highest Shannon indices (1.76 and 1.92) distributed with 0.53 and 0.58 evenness, respectively. A majority (87%) of the Alphaproteobacteria phosphonate producers are Pelagibacterales bacterium with other notable taxa including Bacteria: *Candidatus Actinomarinaceae, Prochlorococcus* sp., *Synechococcus* sp., *Nitrosococcus* sp., and MG-I Archaea: *Candidatus Nitrosomarinus catalina*, *Nitrosopumilus maritimus*, alongside other unidentified Crenarchaeota and Thaumarchaeota genomes.

The gene *phpC* was found in less than half the number of genomes than *pepM* and *aepY*, and encoded by fewer classes in both the general database (47) and marine subset (10). In the full databases, the distribution of retrieved *phpC* sequences are similar to *pepM* and *aepY* with respect to taxonomic ranking, Shannon index (2.49), and evenness (0.60) (Fig. 2A–C, Supplementary Table 5). Within the marine subset, *phpC* has less Shannon index (1.61) but greater evenness (0.61) than the marine subset of *pepM* and *aepY*. All three upstream phosphonate production genes (*pepM*, *aepY*, *phpC*) are found together within Pelagibacterales bacterium, *Prochlorococcus* sp., Thaumarchaeota, and Crenarchaoeta alongside other taxa such as *Oceanospirillales* sp., *Arenimonas donghaensis*, *Desulfuromusa kysingii*, and *Cellulosilyticum lentocellum*.

**Fig. 3:**
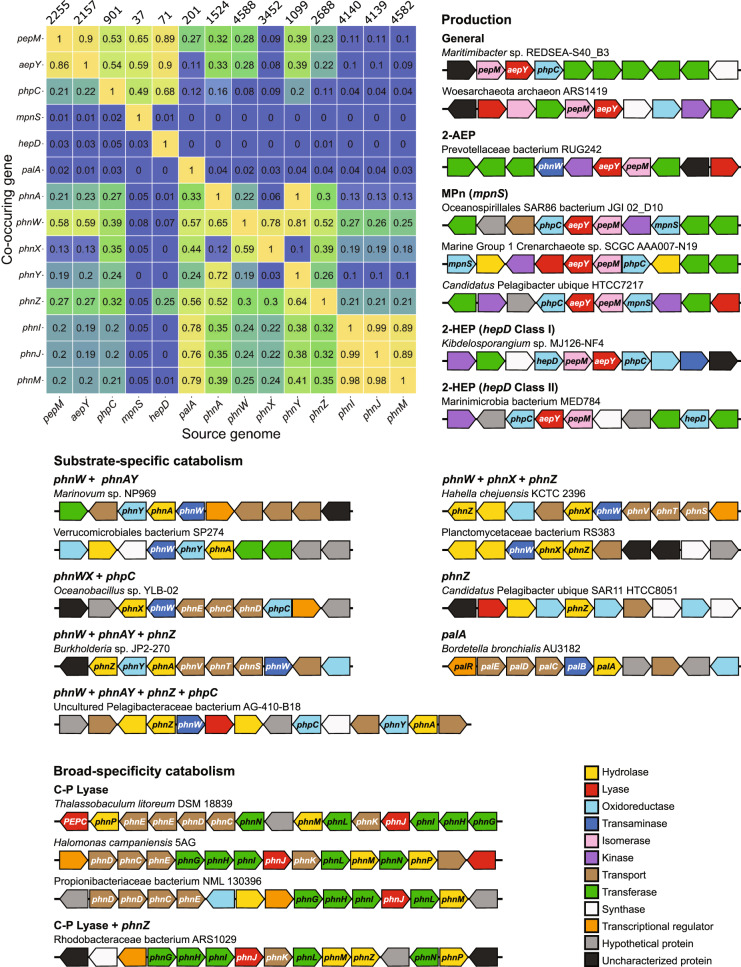
Co-occurrence of phosphonate cycling genes within the same genome and examples of genetic organization of phosphonate cycling genes. The heatmap displays co-occurrence of phosphonate cycling genes. Each column represents the subset of all genomes which contain the source gene and the heatmap value represents the fraction of the source genomes which also contain the co-occurring gene. Heatmap values are not symmetrical due to differing number of genomes represented in each column, database size listed above each column. Examples for phosphonate cycling genomic neighbourhoods were chosen to maximize diversity in synteny with examples from both Bacteria and Archaea where applicable. Several phosphonate-specific ABC transport system clusters are labelled as follows: phnC = phosphonate transport system ATPase; phnD = phosphonate transport system substrate-binding; phnE = phosphonate transport system permease; phnS = 2-AEP transport system substrate-binding; phnT = 2-AEP transport system ATP-binding; phnV = 2-AEP transport system permease; palC = transport system permease; palD = transport system ATP-binding; palE = transport system permease. Genes are colour coded by: red = lyase; orange = transcriptional regulator; yellow = hydrolase; green = transferase; light blue = oxidoreductase; dark blue = transaminase; purple = kinase; pink = isomerase; brown = transport; white = synthase; black = uncharacterized protein; grey = unknown.

We further investigated the relationship between *pepM*, *aepY*, and *phpC* by examining co-occurrence in genomes and synteny with the general, redundant databases. The first two steps in phosphonate biosynthesis are intimately linked (Fig. 3). Out of all genomes with *pepM*, 86% have *aepY*, and out of all genomes with *aepY*, 90% have *pepM*. By contrast, *phpC* is not as closely tied to *pepM* and phosphonate production. We found *phpC* in just over 20% of genomes with the capability of phosphonate production (Fig. 3), implying that a majority of bacterial and archaeal phosphonate production stops at the production of phosphonoacetaldehyde or 2-AEP (Fig. 1A). Furthermore, half of the *phpC* genes were not associated with phosphonate production, given 53% of genomes with *phpC* did not have *pepM* and 54% did not have *aepY* (Fig. 3). In these instances, microbes may use *phpC* within a 2-AEP substrate-specific catabolism operon (Fig. 3) that allows phosphonate compounds to be synthesized by transforming 2-AEP with *phnW* and *phpC* into 2-HEP (Figs. 1A and 3). By repurposing 2-AEP, individuals can still create the specific compound needed while bypassing the energetically unfavourable first step of phosphonate production.

A narrow but diverse selection of taxa encoded MpnS, the marker gene for Mpn production and a key determinant in marine methane production. We observed distinct clades of this enzyme in autotrophic archaea and heterotrophic bacteria (Fig. 2B, C). Within the marine ecosystems, Pelagibacterales, Rhodospirillales, Rickettsiales, Oceanospirillales, Flavobacteriales, and Synechococcales are bacterial candidates for MPn production alongside Thaumarchaeota and Crenarchaeota archaeon (Fig. 2B, C). While six of the bacterial genomes with MpnS also encoded genes for phosphonate catabolism, none of the archaeal MPn producers showed capacity for catabolism (Supplementary Dataset S1). The genomic neighbourhoods for general phosphonate production (*pepM*, *aepY*, *phpC*) and MPn production (*mpnS*) in both bacteria and archaea include genes such as glycosyltransferase, lipopolysaccharide choline phosphotransferase, choline kinase, adenylyltransferase, and arylsulfatase A (Fig. 3) suggesting the potential for synthesis of (methyl)phosphonate esters [93]. This is consistent with previous analysis [29] of the *Nitrosopumilus maritimus SCM1* MPn production genomic neighbourhood and biophysical evidence that MPn producing archaea synthesize an exopolysaccharide modified with MPn similar to 2-AEP modified polymers.

Contrary to the diversity of the other phosphonate production databases, the *hepD* database has low Shannon index (0.62) and evenness (0.45) with 79% of sequences mapping to Actinomycetia including Streptomycetales and Corynebacteriales (Fig. 2B, C). The marine subset has lower Shannon index (0.28) and evenness (0.41) where all sequences derive from Pelagibacterales except one from *Prochlorococcus* sp. The genomic neighbourhood of HMP production may contain genes for cell surface modification such as acetyltransferase, peptidoglycan biosynthesis, and adenylylsulfate transferase, suggesting that some organisms may use HMP as a conjugate for membrane-associated or exported macromolecules similar to theories on MPn utilization. Other examples of *hepD* synteny contain more specific genes such as the HMP dehydrogenase or other enzymes for downstream modification (Fig. 3).

### Marine proteobacteria encode genes for substrate-specific and broad-specificity phosphonate catabolism

Genes for marine substrate-specific phosphonate catabolism were widespread among Proteobacterial classes, and to a lesser extent amongst other classes including Bacilli, Planctomycetes, and Synechococcus (Fig. 2E,F). Marine substrate-specific catabolism has lower average Shannon index (1.00) and evenness (0.43) than the three general production genes (*pepM, aepY, phpC*). The most widespread of these genes was *phnW*, likely due to its pivotal role in 2-AEP transformations as a precursor reaction to *phnAY* or *phnX* (Fig. 1, Supplementary Table 5). Marine hydrolases for 2-AEP catabolism, *phnA*, *phnX*, and *phnZ*, have similar Shannon indices (mean: 1.11 ± 0.05) and evenness (mean: 0.41 ± 0.03) (Fig. 2E, F, Supplementary Table 5).

While not exclusive, sequenced references demonstrate a strong taxonomic partition between Proteobacterial classes for 2-AEP catabolism pathways *phnAWY* and *phnWX*. Over 74% of marine genomes with *phnAWY* are Alphaproteobacteria, in particular Rhodobacterales species such as *Roseovarius nubinhibens*, *Marivita geojedonensis*, and *Pelagicola litoralis*. On the contrary, ~80% of marine genomes with *phnWX* are Gammaproteobacteria, specifically of Vibrionales, Oceanospirillales, and Alteromonadales including a wide range of species from *Vibrio*, *Photobacterium*, *Marinobacterium*, *Halomonas*, and *Pseudoalteromonas*.

Taxonomic distribution for marine *phnZ* was 72% Alphaproteobacteria with Pelagibacterales making up 45% of marine *phnZ* sequences. Note that *phnZ* has the most (17) reference sequences from marine Cyanobacteriia, specifically *Prochlorococcus* sp., than any other phosphonate catabolizing gene. Lack of marine sequence representatives for catabolism of phosphonopyruvate by *palA* suggests that either the substrate is uncommon, therefore the function unnecessary, or marine microbes have other methods of catabolizing phosphonopyruvate, perhaps by the C-P lyase. Overall taxonomic distribution of phosphonate substrate-specific catabolism, specifically targeting 2-AEP, suggests said function is essential to many marine heterotrophs within Alphaproteobacteria and Gammaproteobacteria. However 2-AEP catabolism appears to be less universally important than phosphonate production to marine microbial life since the required genes are found in a less diverse selection of taxa.

Genetic organization for substrate-specific catabolism genes, particularly those targeting 2-AEP, varied widely in line with the numerous options for 2-AEP catabolism (Fig. 3). Though some bacteria specialize in a single 2-AEP degradation pathway such as only containing *phnWAY*, others contained multiple hydrolases for 2-AEP catabolism with some incorporating *phpC* into a 2-AEP specific catabolism operon (Fig. 3). When a genome has two hydrolases for phosphonate catabolism, often *phnZ* was paired with either *phnA* or *phnX*. Co-occurrence between *phnZ* and either *phnA* or *phnX* ranged between 30-50%, whereas co-occurrence between *phnA* and *phnX* was between 6-12% (Fig. 3). This discrepancy in co-occurrence may be due to the metabolic similarity between *phnA* and *phnX*, where having both may be redundant. Both of these enzymes rely on *phnW* for 2-AEP catabolism and produce carbon metabolites, whereas *phnZ* does not need *phnW* and produces the amino acid glycine (Fig. 1B).

C-P lyase genes representing substrate non-specific catabolism were overwhelmingly attributed to Alphaproteobacteria which consisted over 75% of all collected marine sequences for *phnIJM* (Fig. 2H, I). A wide variety of Rhodobacterales, spanning 55 different genus are the most numerous representatives, followed by Pelagibacterales and Rhodospirillales. The genes in all three databases have very high genome co-occurrence, 89–99%, as expected given all three operate within the same enzyme complex (Fig. 3). Gene co-occurrence, Shannon index, and evenness is lower for *phnM* than the other two C-P lyase components, *phnI* and *phnJ*, likely due to instances of organisms containing two copies of *phnM* where one copy lies outside the C-P lyase operon [94]. C-P lyase gene databases have lower Shannon index (mean 0.81 ± 0.11) than phosphonate production and 2-AEP substrate-specific catabolism genes (*phnAWXZ*) (Fig. 3D, G), suggesting broad-specificity phosphonate catabolism by the C-P lyase is a narrowly distributed function (Supplementary Table 5). Organization of C-P lyase operons held the most consistency between example genomes, likely due to the high number of genes simultaneously utilized for lyase construction. These operons encoded a consecutive string of lyase subunits, including a generic phosphonate transporter (*phnCDE*) and GntR transcriptional regulator (Fig. 3). C-P lyase genes had low genomic co-occurrence with all other phosphonate cycling genes with notable co-occurrence between *phnW* at 26%, *phnZ* at 21%, and *phnX* and 18% (Fig. 3). The low rate of co-occurrence may be due to redundancy in function for P harvesting between the C-P lyase and substrate-specific catabolism. In some cases there are instances of a substrate-specific hydrolase gene located within the C-P lyase operon (Fig. 3).

### Phosphonate biosynthesis genes are globally prevalent in oceans and increase in mesopelagic waters

Following curation of *phn*-gene databases, we analysed 121 metagenomes and 91 metatranscriptomes from the publicly available TARA Oceans expedition (spanning samples from the Atlantic, Indian, Pacific, and Southern Ocean and Red Sea) to investigate the global potential for marine phosphonate cycling. Measuring the proportion of the community capable of performing specific tasks through metagenomics indicates the long-term selective pressures that shape P-cycling and microbial communities.

**Fig. 4:**
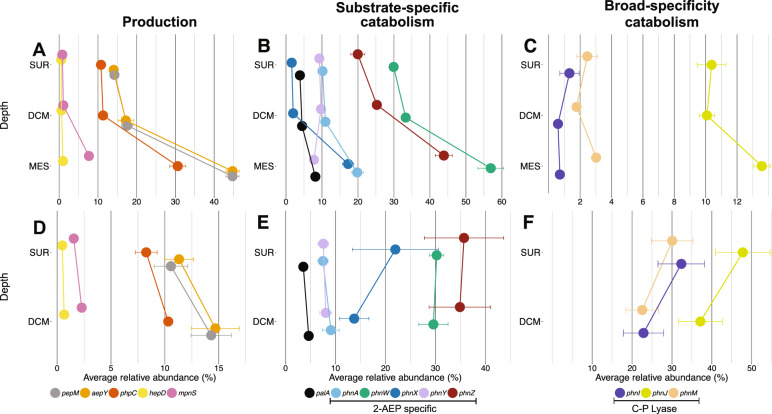
Global distribution of phosphonate cycling genes across TARA Oceans metagenomes from different depths. Results from DIAMOND searches of phosphonate cycling gene databases against the TARA Oceans Project. Gene hits were normalized by copy number and sequence length with relative abundance calculated based on the length-normalized average of five conserved single-copy genes as a proxy for a count of individuals within a sample, displayed as a relative percentage of community members. Results are shown for selected genes representing phosphonate (**A**, **D**) production, (**B**, **E**) substrate-specific catabolism, and (**C**, **F**) broad-specificity catabolism. Relative abundance is shown separately for a global model (**A**–**C**) and Mediterranean Sea (**D**–**F**). Results are shown for surface (SUR), deep chlorophyll maximum (DCM), and, for the global model, mesopelagic (MES) zones. Results from ANOVA and Tukey HSD analysis comparing gene abundance between genes, depths, and geographic regions can be found in Supplementary Tables S6 and S7.

Potential for phosphonate production (*pepM*) was globally ubiquitous across all depths, with 14–17% of the community encoding in the surface waters and deep chlorophyll maximum (DCM), increasing to 45% in mesopalagic waters (Fig. 4A, Supplementary Tables S6 and S7), highlighting the importance of phosphonate compounds to marine microbial communities. Relative abundance of *phpC* was 64–76% that of *pepM* and *aepY* across all depths (Fig. 4A). We observed significant increase in relative abundance between the surface and mesopelagic for *pepM* (ANOVA: *F* = 1262, *p* < 0.001; Tukey HSD *p* < 0.001), *aepY* (ANOVA: *F* = 1358, *p* < 0.001; Tukey HSD *p* < 0.001), and *phpC* (ANOVA: *F* = 272.4, *p* < 0.001; Tukey HSD *p* < 0.001) suggesting a greater need for phosphonate production at depth. In contrast, the relative abundance of the genes to produce Mpn (*mpnS*) and hydroxymethylphosphonate (*hepD*) were each found to be present in <1.5% of surface and DCM communities. The abundance of both *mpnS* (ANOVA: *F* = 517.2, *p* < 0.001; Tukey HSD *p* < 0.001) and *hepD* (ANOVA: *F* = 62.99, *p* < 0.001; Tukey HSD *p* < 0.001) significantly increased in the mesopelagic, up to 7.7% of the community for *mpnS* (Fig. 4A, supplementary Tables S6 and S7), though both genes stayed below 0.25 transcripts per 100,000 reads of the metatranscriptome at all depths (Supplementary Fig. S7).

Changes in taxa harbouring phosphonate production genes echoed the significant increases in production potential between depths. Pelagibacterales reads dominated surface production and remained present in the mesopelagic. Increased relative abundance of phosphonate production genes in the mesopelagic was attributed to populations of Thaumarchaeota and Crenarchaeota phosphonate producers, particularly for *mpnS* (Supplementary Fig. S8). Other phosphonate producers from Actinomycetia such as Candidatus Actinomarinales and Streptosporangiales alongside Candidatus Marinimicrobia were present at all depths. A breakdown of taxonomy for each gene by sample can be found in Supplementary Table S8.

### Substrate-specific catabolism of 2-AEP is the dominant global strategy for phosphonate catabolism at all depths

Genes for 2-AEP substrate-specific phosphonate catabolism (*phnAWYZ*) were widespread throughout the TARA Oceans metagenomes, found amongst 9–33% of surface and DCM communities increasing significantly to 8–57% of the mesopelagic community members (*phnA*: ANOVA *F* = 78.62 *p* < 0.001, Tukey HSD *p* < 0.001; *phnW*: ANOVA *F* = 145.4 *p* < 0.001, Tukey HSD *p* < 0.001; *phnY*: ANOVA *F* = 14.03 *p* < 0.001, Tukey HSD *p* < 0.001; *phnZ*: ANOVA *F* = 209.7 *p* < 0.001, Tukey HSD *p* < 0.001) (Fig. 4B). The most widespread strategy for 2-AEP catabolism was *phnZ* as *phnW* serves an intermediate role in multiple pathways spanning production and catabolism. Both *phnX* and *palA* were found in low relative abundance in surface and DCM communities which, like other phosphonate cycling genes, increased significantly from the DCM to mesopelagic (*palA*: ANOVA *F* = 36.36 *p* < 0.001, Tukey HSD *p* < 0.001; *phnX*: ANOVA *F* = 385.6 *p* < 0.001, Tukey HSD *p* < 0.001) (Fig. 4B). Preference for different metabolic products aside from phosphate, namely production of acetate (*phnA*), glycine (*phnZ*), or acetaldehyde (*phnX*) (Fig. 1B), may determine the observed difference in relative abundance.

Most higher relative abundance downstream consumption genes (*phnAWYZ*) mapped to Alphaproteobacteria, specifically Pelagibacterales, Bradyrhizobiales, Rhodobacterales, and Rhodospirillales, but read taxonomy diversified in the mesopelagic to include Desulfobacterales, Acidiferrobacterales, Alteromonadales, Neisseriales, Burkholderiales, and Candidatus Marinimicrobia (Supplementary Fig. S9). Taxonomic assignment of *phnX* featured greater representation of Bacilli (*Enterococcus* sp. and *Secundilactobacillus* sp.) and Gammaproteobacteria (Acidiferrobacterales) (Supplementary Fig. S9).

The relative abundance of broad-specificity C-P lyase genes (*phnIJM*) was significantly lower than the marker gene for phosphonate production (*pepM*) (*phnI*-*pepM* Tukey HSD: SUR *p* < 0.001, DCM *p* < 0.001, MES *p* < 0.001; *phnJ*-*pepM* Tukey HSD: SUR *p* < 0.12, DCM *p* < 0.001, MES *p* < 0.001; *phnM*-*pepM* Tukey HSD: SUR *p* < 0.001, DCM *p* < 0.001, MES *p* < 0.001) and common 2-AEP specific hydrolases (*phnAZ*) (*phnI*-*phnA* and *phnI-phnZ* Tukey HSD: SUR *p* < 0.001, DCM *p* < 0.001, MES *p* < 0.001; *phnJ*-*phnA* and *phnJ-phnZ* Tukey HSD: SUR *p* < 0.001, DCM *p* < 0.001, MES *p* < 0.001; *phnM*-*phnA* and *phnM-phnZ* Tukey HSD: SUR *p* < 0.001, DCM *p* < 0.001, MES *p* < 0.001). We estimated *phnI*, *phnJ*, and *phnM* were encoded by between 1.3–10.4% and 0.7–13.6% of global marine microorganisms in the surface and mesopelagic, respectively (Fig. 4C). No significant difference in relative abundance was observed between *phnI* and *phnM* whereas relative abundance of *phnJ* was always significantly higher in the surface (ANOVA: *F* = 401.3, *p* < 0.001; Tukey HSD *phnI*-*phnJ*: *p* < 0.001, *phnI-phnM: p* < 0.999, *phnJ-phnM: p* < 0.001), DCM (ANOVA: *F* = 254.6, *p* < 0.001; Tukey HSD *phnI-phnJ: p* < 0.001, *phnI-phnM: p* < 1, *phnJ-phnM: p* < 0.001), and mesopelagic (ANOVA: *F* = 676.3, *p* < 0.001; Tukey HSD *phnI-phnJ: p* < 0.001, *phnI-phnM: p* < 0.999, *phnJ-phnM: p* < 0.001) (Fig. 4) (Supplementary Tables 6, 7).

The relative abundance of *phnI* (ANOVA: *F* = 26.24, *p* < 0.001; Tukey HSD: *p* < 0.001) significantly decreased between surface and mesopelagic communities while *phnJ* (ANOVA: *F* = 8.651, *p* < 0.001; Tukey HSD: *p* < 0.001) and *phnM* (ANOVA: *F* = 34.29, *p* < 0.001; SUR-MES Tukey HSD: *p* < 0.001) significantly increased between depths (Fig. 4C, Supplementary Tables S6 and S7). However, the change in relative abundance for *phnI* (−38%), *phnJ* (+31%), and *phnM* (+23%) was minor compared to the influx of phosphonate cycling genes that showed 2-fold (*palA, phnAWZ*), 3-fold (*pepM*, *aepY*), and 10-fold (*mpnS*, *phnX*) increases in relative abundance from the surface to mesopelagic (Fig. 4).

Taxonomic assignment to C-P lyase reads was narrow and changed little between depths, despite inconsistencies between the three genes. Whereas *phnI* mapped to alphaproteobacterial mixo/heterotrophs (Pelagibacterales, Rhodospirillales, Rhodobacterales) relevant to the marine environment (Supplementary Fig. S9), neighbouring gene *phnJ* mapped to several different orders of Actinomycetia (Propionibacteriales, Micrococcales, Corynebacteriales) with a minority of alphaproteobacterial reads matching *phnI* (Pelagibacterales, Rhodospirillales, Rhodobacterales) (Supplementary Fig. S9). Reference taxonomy mapping for *phnM* reflected both the Alphaproteobacteria of *phnI* and Actinomycetia of *phnJ* (Supplementary Fig. S9).

C-P lyase genes in the surface may reflect a consistent availability of organic phosphonates other than 2-AEP. Surface catabolism of MPn by the C-P lyase is the primary avenue for aerobic methane production within the oceanic methane paradox model. Assuming turnover of only a small fraction of the MPn inventory [28] for any site is enough to affect local atmospheric methane levels, we predict this process can be driven by a small proportion of the community specifically aforementioned Alphaproteobacteria and Actinomycetia families. Given the significantly increased potential for phosphonate production in the mesopelagic coupled with the transfer of increasingly recalcitrant phosphonates from the surface to the deep by preferential remineralization of sinking particles [12], we expected to find greater relative abundance for broad-specificity catabolism by the C-P lyase in the deep with larger increases in relative abundance with depth than observed.

Overall, 2-AEP substrate-specific catabolism by *phnZ* (Fig. 1B) is the dominant strategy for phosphonate remineralisation with relative abundances reaching 2- to 14- fold greater than all other catabolism genes (Fig. 4) and up to 3-fold the number of transcripts (Supplementary Fig. 7). While *phnW* is observed with higher relative abundance and transcription than *phnZ*, it is used in multiple catabolism and production pathways. We hypothesize that substrate-specific catabolism strategies specifically targeting 2-AEP are the most generically useful strategy for phosphonate catabolism, offering the greatest survival potential due to the reliably accessible substrate providing opportunity for C and N assimilation alongside harvesting P. Due to lower relative abundance and transcripts, we hypothesize the C-P lyase is a niche function for specialist heterotrophs targeting DOM or fast-growing individuals with energy supplementation that need to meet higher P demands for growth and replication.

### Deviation from global trends under extreme nutrient limitation

Following the global analysis, we separately analysed Mediterranean Sea (MED) samples from the TARA Expedition, which is known to be an extremely P-limited system [95, 96]. In our selection of samples, the Mediterranean samples had the lowest average phosphate in the surface at 0.01 µmol/L and the second lowest average phosphate in the DCM at 0.03 µmol/L (Supplementary Table 3). These metagenomes provided a unique opportunity to test whether trends of phosphonate cycling gene abundance and taxonomy established by the global survey reflect variations in P availability. We chose to examine the Mediterranean alone as it displayed the most extreme changes from the global dataset.

Compared to the global dataset, Mediterranean communities had the lower relative abundance for general phosphonate production (*pepM*) in the surface (ANOVA: *F* = 37.17, *p* < 0.001) and DCM (ANOVA: *F* = 8.51, *p* value > 0.004) (Fig. 4D, Supplementary Table S6). However, based on *mpnS* abundance the potential for Mpn production in the Mediterranean Sea was approximately twofold higher than in the surface global oceans (ANOVA, *F* = 21.38, *p* < 0.001) in the DCM (ANOVA, *F* = 39.03, *p* < 0.001) (Fig. 4B).

We originally hypothesized that microbes with diminished access to phosphorus may need to prioritize other functions necessary for growth and survival over phosphonate production, though in regards to MPn the observed trends contradict this expectation. With the significant increase in *mpnS* compared to the global setting, the model of MPn as long-term phosphorus storage for bacteria seems realistically applicable to the Mediterranean setting. It is notable that the overwhelming majority of Mediterranean *mpnS* reads map to several species of Pelagibacterales, which was the model organism for the observation of P-granule production when grown on MPn [97].

Among the phosphonate catabolism enzymes, the C-P lyase is one of the most common pathways for phosphonate catabolism in the Mediterranean ranging from 30–47% relative abundance in the surface and 22–37% relative abundance in the DCM. Relative abundance of *phnI* (SUR 24-fold increase, ANOVA: *F* = 408.2, *p* < 0.001; DCM 39-fold increase, ANOVA: *F* = 211.2, *p* < 0.001), *phnJ* (SUR 4.5-fold increase, ANOVA: *F* = 193.2, *p* < 0.001; DCM 3.5-fold increase, ANOVA: *F* = 90.84, *p* < 0.001), and *phnM* (SUR 12-fold increase, ANOVA: *F* = 723.3, *p* < 0.001; DCM 13-fold increase, ANOVA: *F* = 475.4, *p* < 0.001) all significantly increased compared to the global dataset in stark contrast to the minimal potential of broad-specificity catabolism in the global ocean metagenomes (Fig. 4E, F). The dominant substrate-specific catabolism gene from the global TARA dataset, *phnZ*, retained the highest of all substrate-specific catabolism gene relative abundances in the Mediterranean. On the other hand, *phnAY* significantly decreased in relative abundance in the surface (*phnA* ANOVA: *F* = 7.112, *p* < 0.008; *phnY* ANOVA: *F* = 4.314, *p* < 0.038) with no significant change in the DCM (*phnA* ANOVA: *F* = 2.312, *p* < 0.128; *phnY* ANOVA: *F* = 2.798, *p* < 0.0944) (Fig. 4B, E). Opposed to the global oceans, *phnX* had substantially increased relative abundance in both the surface (13-fold increase, ANOVA: *F* = 167.9, *p* < 0.001) and DCM (6-fold increase, ANOVA: *F* = 152.1, *p* < 0.001) in Mediterranean microbial communities (Fig. 4D).

Alongside inconsistencies in gene abundance patterns in the Mediterranean Sea compared to the global oceans, our findings indicate phosphonate cycling in the Mediterannean is more taxonomically restricted than observed in the global TARA dataset. Pelagibacterales remained the largest contributors to all facets of phosphonate cycling from production to catabolism in the Mediterranean Sea. Other families such as Rhodospirillales, Rhodobacterales, and Rhizobiales were well represented amongst phosphonate catabolism genes. The main differences in taxonomy relates to *phnX* and *phnIJM* where genes from Bacilli, Deltaproteobacteria, Gammaproteobacteria, and Actinomycetia observed in the global TARA dataset were absent from the Mediterranean samples (Supplementary Fig. S9, Supplementary Table S8).

We expect the lyase’s role in global oceans to operate differently than in an environment of extreme P limitation like the Mediterranean Sea. The low relative abundance of C-P lyase genes from the global TARA dataset indicates generalized phosphonate catabolism as a niche, specialist function (Fig. 4). However, when the environment severely lacks phosphate, microbes will be more reliant on organic forms of phosphorus to meet their needs. If an individual restricts themselves to substrate-specific catabolism of 2-AEP, they may miss opportunities to obtain phosphorus from other organic compounds which could be detrimental to short-term survival. Therefore, we hypothesize that generalized organic phosphonate scavenging by the C-P lyase becomes a common strategy for P acquisition when overcoming extreme phosphate limitation due to the flexibility of substrate targets.

### Depth and seasonality underpin composition of phosphonate cycling community

We determined the seasonal variability in phosphonate cycling potential by analysing 21 new metagenomes from the Munida Time Series Transect (MOTS). This local 65 km transect provides insight to an under sampled region (Southern Ocean) in the TARA Oceans Project, spanning contrasting water masses in a short distance offshore: surface sub-tropical (STW), surface sub-Antarctic (SAW), and 500 m deep sub-Antarctic (SAW-MES). These sites have divergent nutrient conditions between the macronutrient-limited STW and the high-productivity, low-chlorophyll micronutrient-limited SAW. Metagenomes were analysed from samples collected in summer and winter from 2014 to 2017.

**Fig. 5:**
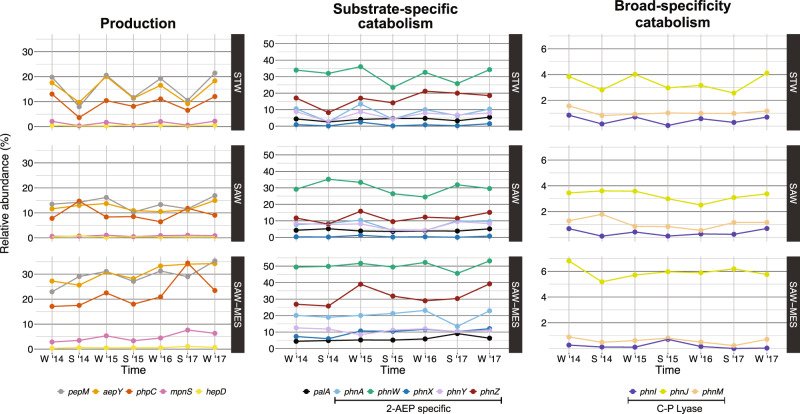
Temporal distribution of phosphonate cycling genes across Munida Time Series Transect (MOTS). Results from DIAMOND searches of phosphonate cycling gene databases against the MOTS dataset. Gene hits were normalized by copy number and sequence length with relative abundance calculated based on the length-normalized average of five conserved single-copy genes as a proxy for a count of individuals within a sample, displayed as a relative percentage of community members. Abundance data is shown for select dates between June 2014 and June 2017, where June/August samples represent winter (W) and December/February time points represent summer (S). Results are shown for marker genes for phosphonate production (left), substrate-specific catabolism (center), and broad-specificity catabolism (right). Data for the three water masses are shown separately for sub-tropical (STW; top), sub-Antarctic (SAW; middle), and 500 m deep sub-Antarctic (SAW-MES; bottom) samples. Results from the ANOVA analysis comparing seasonal relative gene abundance can be found in Supplementary Table S9.

Phosphonate cycling gene relative abundance was consistent with patterns observed in TARA Oceans samples (Fig. 5). Considering the average relative abundance across time for both surface water masses (STW and SAW), relative abundances for general phosphonate production: *pepM* (19.6%), *aepY* (19.1%), and *phpC* (13.6%), were 6- to 39-fold higher than that of specialized downstream production genes: *mpnS* (2.3%) and *hepD* (0.5%) (Fig. 5). The relative abundance for the most common genes for 2-AEP substrate-specific catabolism observed in the TARA dataset: *phnW* (37.1%) and *phnZ* (20.1%), were 5- to 123-fold higher than that of broad-specificty catabolism by C-P lyase: *phnI* (0.3%), *phnJ* (4.2%), and *phnM* (0.9%), which retained low presence in the communities (Fig. 5). Similar to the TARA analysis, transitioning into deep mesopelagic waters from SAW to SAW-MES resulted in enrichment of all phosphonate cycling genes except *hepD* and *phnI* (ANOVA: *pepM* *F* = 85.05, *p* < 0.001; *aepY* *F* = 154.18, *p* < 0.001; *phpC* *F* = 24.49, *p* < 0.001; *mpnS* *F* = 35.04, *p* < 0.001; *hepD* *F* = 1.17, *p* < 0.305; *palA* *F* = 6.11, *p* < 0.029; *phnA* *F* = 59.32, *p* < 0.001; *phnW* *F* = 138.49, *p* < 0.001; *phnX* *F* = 115.17, *p* < 0.001; *phnY* *F* = 15.989, *p* < 0.002; *phnZ* *F* = 72.70, *p* < 0.001; *phnI* *F* = 1.32, *p* < 0.272; *phnJ* *F* = 126.98, *p* < 0.001; *phnM* *F* = 7.59, *p* < 0.017) (Fig. 5) (Supplementary Table S9).

Along with relative gene abundance, the taxonomy of genes found in MOTS reflects the taxonomic distributions established from the TARA samples (Supplementary Fig. S11). Alphaproteobacteria (Pelagibacterales, Rhodobacterales) and MG-I Archaea (Thaumarchaoeta, Crenarchaeota) dominate phosphonate production across the spatiotemporal gradient. The proportion of all *pepM* genes in surface communities that map to Alphaproteobacteria range from 61–66% in the summer and 36–47% in the winter while Thamarchaeota and Crenarchaeota *pepM* in surface waters is limited to STW winter making up 11.9% and 5% of surface *pepM*. Even in the mesopelagic 17–26% of *pepM* maps to Alphaproteobacteria whereas 23% and 11.5% map to Thaumarchaeaota and Crenarchaoeta, respectively.

A majority of substrate-specific (6–98%) and broad-specificity catabolism (13–97%) genes map to Alphaproteobacteria (Pelagibacterales, Rhodospirillales, Rhodobacterales) depending on watermass and season. Genes *phnAY* diversify in mesopelagic waters to include Deltaproteobacteria (Desulfarculales, Myxococcales, unclassified) and Gammaproteobacteria (Acidiferrobacterales, Oceanospirillales). However, *phnX* shows broad taxonomic diversity across all watermasses and seasons to include Gammaproteobacteria (Acidiferrobacterales, Vibrionales), Planctomycetia (Planctomycetia), and Deltaproteobacteria (unclassified, Desulfovibrionales). A breakdown of taxonomy for each gene by sample can be found in Supplementary Table S10.

Comparing gene abundance across four years revealed a clear seasonal cycle where genes for phosphonate production and consumption demonstrated increased relative abundance during winter months (June–August). We observed the strongest seasonal effect in the STW, where relative abundance of ten phosphonate cycling genes significantly increased from summer to winter (Fig. 5, Supplementary Table S9) [listed as (% increase, ANOVA *F*-value, ANOVA *p* value): *pepM* (102%, *F* = 92.26, *p* < 0.0002); *aepY* (80%, *F* = 60.22, *p* < 0.0005); *phpC* (91%, *F* = 18.62, *p* < 0.008); *mpnS* (323%, *F* = 125.98, *p* < 0.0001); *phnW* (26%, *F* = 9.78, *p* < 0.026); *phnA* (151%, *F* = 25.902, *p* < 0.003); *phnY* (81%, *F* = 13.58, *p* < 0.014); *phnX* (720%, *F* = 7.61, *p* < 0.040); *phnI* (334%, *F* = 38.277, *p* < 0.0016); *phnJ* (36%, *F* = 13.594, *p* < 0.014)]. In contrast, the number of genes showing seasonal variation from summer to winter decreased to two when transitioning into the SAW [listed as (% increase, ANOVA *F*-value, ANOVA *p* value): *phnZ* (41%, *F* = 7.38, *p* < 0.042); *phnI* (265%, *F* = 8.16, *p* < 0.036)] (Supplementary Table S9) with no genes demonstrating significant seasonal dynamics in the mesopelagic.

The changes in phosphonate production over space and time were associated with the availability of thaumarcheotal phosphonate producers (Supplementary Fig. S11). In the surface waters of STW, the aforementioned twofold increase in general phosphonate production potential (pepM) and 442% increase in MPn production potential during winter is also linked to the influx of Archaea capable of phosphonate production (Supplementary Fig. S11). While it is unknown what drives seasonality of such archaea in surface waters, this phenomenon has been reported in previous studies [98–101] and is potentially linked to the breakdown of stratification during winter months, resulting in upwelling from the mesopelagic to surface ocean [102]. We hypothesize that surface seasonality of phosphonate production is a global phenomenon tied to the seasonal vertical movement of Thaumarchaeota, which may also drive observed seasonal changes in *phnIJ* relative abundance by providing more substrate for lyase activity. Seasonal potential for MPn production may also imply seasonal potential for methane production within the methane marine paradox model where methane release into the atmosphere increases either during or lagging behind the months of increased surface populations of Thaumarchaeota.

We performed an NMDS based on Bray-Curtis dissimilarity (Stress = 0.049) on the results of the phosphonate gene metagenomic screen to determine the most important factors potentially driving relative abundance and taxonomic composition of the community harbouring phosphonate cycling genes (Supplementary Fig. S12). The MOTS system demonstrates a hierarchy of deterministic factors regarding the composition and taxonomy of phosphonate cycling genes within a community, which can be seen by the distribution of points along the NMDS1 axis (Supplementary Fig. S12). Depth (ANOSIM: *R* = 0.8548, *p* < 0.001) is the most important parameter demonstrated by the SAW-mesopelagic samples clustering separately from the STW/SAW surface samples (Supplementary Fig. S12). We then excluded SAW-MES samples to identify the deterministic factor of the phosphonate cycling communities in surface waters and found that seasonality (ANOSIM: *R* = 0.8285, *p* < 0.001) has a greater effect than water mass (ANOSIM: *R* = 0.0632, *p* < 0.19) on the composition and taxa of phosphonate cycling potential in surface waters. Phosphonate cycling communities from the STW/SAW surface were more alike each other in the same season than they were to themselves at different time points in the year, despite the opposing nutrient conditions in both water masses. Phosphonate cycling gene abundance and composition in the mesopelagic more closely reflects the winter surface community’s functions rather than the summer community, in part due to the ubiquitous presence of MG-I archaea in the mesopelagic and their appearance in surface waters during the winter.

### Phosphonate cycling genes relationship with environmental Pi shifts between surface and deep

**Fig. 6:**
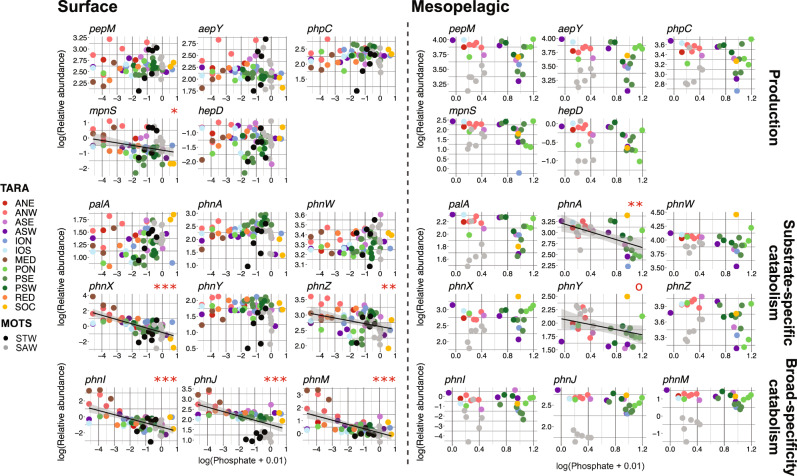
Linear regression between phosphonate cycling gene relative abundance and environmental phosphate concentration. Results from a series of linear regressions between log-transformed length-normalized/copy-normalized relative gene abundance and log-transformed environmental phosphate concentrations for all MOTS and TARA samples from the surface and mesopelagic with available metadata. Surface analysis is shown on the right while mesopelagic analysis is on the left starting with production genes across the top, substrate-specific genes in the middle, and broad-specificity C-P lyase genes along the bottom. Circles are coloured based upon source sample region. Slope significance is indicated by red text above the top right corner of each individual graph such that: ‘o’ if *p* < 0.1; ‘*’ *p* < 0.05; ‘**’ *p* < 0.01; ‘***’ *p* < 0.001.

Finally, we investigated the connection between environmental Pi concentrations and phosphonate cycling gene relative abundance by linear regression using both the TARA and MOTS datasets. In surface waters, upstream phosphonate production (*pepM*, *aepY, phpC*) had no significant relationship to environmental Pi (Supplementary Table S11), showing the potential production of phosphonates is important to retain even within P-limited environments. However, *mpnS* had a significant inverse relation to Pi concentration (Supplementary Table S11), indicating MPn may confer a survival benefit to an individual under P-starvation that cannot be accomplished with other phosphonate compounds (Fig. 6).

The most common catabolism gene, *phnZ*, along with *phnX* and the C-P lyase (*phnIJM*) all displayed significant, inverse relationships with environmental Pi concentrations (Fig. 6, Supplementary Table S11) implying the main role of these three metabolic pathways is P-acquisition. The inverse relationship is best exemplified by the significant increase in relative abundance between the global model and Mediterranean waters (Fig. 4, Supplementary Tables S6, S7). On the other hand, *phnAWY* for 2-AEP specific catabolism showed no relationship with Pi concentrations (Fig. 6, Supplementary Table S11). In the surface, 2-AEP may serve as a well-rounded macronutrient parcel, so if Pi concentrations are high then 2-AEP is still a valuable target substrate for scavenging C/N. We hypothesize that *phnAY* preference for carbon sequestration over *phnX* and *phnZ* is due to the end product being acetate rather than acetaldehyde or glycine (Fig. 1). While 2-AEP scavenging by *phnAWY* could provide a consistent source of macronutrients, we hypothesize that *phnXZ* and the C-P lyase are the prevailing strategies for Pi acquisition from phosphonates under severe Pi-limitation in the surface or reserved for specialist DOP recyclers under normal surface conditions.

The rules change in the mesopelagic where Pi concentrations are much higher. Contrasting results from the surface, *phnAY* were the only two genes to show a significant inverse relationship to Pi (Fig. 6, Supplementary Table S11). The role of these two genes has seemingly shifted from general macronutrient acquisition to Pi scavenging in the face of Pi limitation. Deviating from the trend, *phnW* shows no relation to mesopelagic Pi concentrations. We hypothesize that *phnW* use for phosphonate production increases in the deep ocean, following the significant increase in upstream phosphonate biosynthesis genes in the mesopelagic. In environments with more available Pi, we expect individuals to take the path of least resistance, favouring Pi assimilation over expending energy and resources for DOP catabolism. Increased ambient abundance of Pi may also lessen the strain on individuals to meet P-demands, allowing for more individuals to engage in phosphonate production. We observed this effect in both the TARA and MOTS analysis where potential for phosphonate production was significantly elevated in the mesopelagic (Figs. 4, 5) which suggest a larger inventory of phosphonates and phosphonate-conjugated compounds at depth. Given a similar increase in potential for phosphonate catabolism while decoupling from phosphorus pressure, we hypothesize that a large proportion of phosphonate catabolism in the mesopelagic is carried out for P-redox rather than nutrient acquisition.

## Conclusions

This study is the most comprehensive metagenomic/metatranscriptomic survey of marine microbial phosphonate cycling to date, combining the extensive spatial range of the TARA Oceans Project with seasonal measurements from the unique MOTS dataset to evaluate all relevant enzymes involved in oceanic phosphonate biosynthesis and catabolism for surface and deep waters. Phosphonate biosynthesis genes have a strong ubiquitous presence in mesopelagic waters (Figs. 4, 5) with seasonal fluctuation in the surface waters (Fig. 5). Despite the first two steps in phosphonate biosynthesis consistently retaining higher relative abundances than other production genes, potential for MPn biosynthesis was always present in all depths across the globe (Fig. 4) and linked to Alphaproteobacterial and archaeal populations (Supplementary Fig. 8). A consistent source of MPn across the global surface waters can translate to a consistent release of methane gas by phosphonate consumers as MPn cleavage in surface waters is sufficient to constitute a major source of atmospheric methane [28]. Seasonality for archaeal populations in the surface (Supplementary Fig. 11) may translate to seasonal fluctuations of oceanic contributions to atmospheric methane emissions. Further work investigating the role of MPn in the cell and its avenue for release into the environment would enlighten the full process of aerobic production of marine methane.

Marine microbial catabolism of phosphonates divides into two different strategies: substrate-specific catabolism, a majority of which targets 2-AEP, and the breakdown of diverse phosphonates, otherwise recalcitrant to the community, by the C-P lyase. Based on relative gene abundance, 2-AEP—specific catabolism was the primary strategy for phosphonate consumption in all environments and depths, except for the extreme P-deplete waters of the Mediterranean Sea (Fig. 4). 2-AEP is the most abundant phosphonate substrate and a small molecule offering N assimilation alongside P. Under low P conditions organisms will likely take the path of least resistance to obtain P and preferentially remineralize P-esters over phosphonates [12]. However, under high P conditions and particularly when N is limiting, aminophosphonates will be preferentially consumed relative to esters [103] which may be explained by 2-AEP simultaneously serving as a P and N source. Some organisms may cycle 2-AEP regardless of local Pi concentrations through substrate-inducible gene regulation [43]. We suspect 2-AEP catabolism is generically useful to any life-strategy in the marine environment requiring only two or three genes for a lower relative cost of gene retention compared to broad-specificity catabolism while providing a consistent source of essential nutrients.

C-P lyase is uniquely able to catabolize a broad range of phosphonates to yield P. While 2-AEP is the most abundant phosphonate, organisms will inevitably run into other phosphonate compounds and would be unable to take advantage of the encounter without the C-P lyase for P assimilation. Being pho-regulated, the C-P lyase is a unique tool for P scavenging within P-deplete environments such as the Mediterranean Sea. Though even outside of extreme oligotrophy, the relative rarity of the C-P lyase provides a niche for DOM scavengers who gain exclusive access to more recalcitrant sources of P otherwise untouchable by the rest of the community.

However, the exclusive function of C-P lyase comes at a cost. A 14 gene operon encoding a multi-subunit protein complex requires more resources from the cell relative to 2-AEP substrate-specific catabolism. Likewise, microbes that carve out their niche by remineralizing DOM, have a fast-growing lifestyle with high P demand for growth/replication, or live under extreme P limitations where much of the available P will be constrained within rapidly cycled DOM would greatly benefit from the C-P lyase’s broad-specificity phosphonate catabolism. Aerobic anoxygenic photosynthetic mixotrophic bacteria, highly adaptive organisms that compose >10% of the microbial community in some upper ocean waters [104], would be prime candidates for the C-P lyase as seen in some Roseobacter [104, 105]. They can supplement energy through a niche unavailable for other bacteria to offset access to another niche for phosphorus acquisition with similar exclusivity. However, when we looked in environments with extreme P-limitation like the Mediterranean Sea, the C-P lyase became one of the dominant strategies for phosphonate catabolism. Thus, under extreme P-limited conditions, the necessity for survival outweighs the cost of lyase construction.

As a large fraction of marine microbial P is sourced from DOP, it is no surprise that our findings substantiate the widespread importance of phosphonate cycling for microbial communities in the marine environment. High prevalence of enzymes for phosphonate remineralization imply fast turnover of DOP as phosphorus will be continually (re)mineralized. In the mesopelagic waters, this importance is even greater as we observed significant increases in phosphonate cycling genes. The microbial community processes DOP as it sinks, preferentially targeting molecules easier to catabolize like P-esters over the inert C-P bond in phosphonates [12]. When sinking DOP reaches the mesopelagic depths, phosphonates will make-up a greater percentage of the available DOP from sinking particles since less recalcitrant compounds like P-esters will be consumed first. Furthermore, at these depths Pi is more abundant in the environment, lessening the strain on individuals to meet the bare-minimum requirements for survival and replication which we expect gives community members more freedom to use phosphorus for alternative means utilizing phosphonates like structural components. Genes such as glycosyltransferase and choline kinase involved in biosynthesis of membrane or extracellular structures are often found within the genomic neighbourhood of phosphonate production genes (Fig. 3). Thaumarchaeota, members of the ubiquitous archaeal populations in the mesopelagic, have been previously hypothesized to produce phosphonates for conjugates to exopolysaccharides [29, 93] which may be enabled by the increased availability of Pi in deep ocean environments. The hypothesized increased phosphonate inventory in the mesopelagic from the combination of sinking particles and increased production leads to a higher capacity for phosphonate catabolism within the community. However the decoupling of phosphonate catabolism gene abundance from Pi pressure suggests a major proportion of phosphonate catabolism at depth is for energy generation through redox rather than nutrient acquisition.

The contributors to increased relative abundance of phosphonate production genes in the mesopelagic: Preferential remineralization, less strain to meet P-requirements, and large archaeal populations may also contribute to the seasonal aspect of phosphonate cycling observed in MOTS. When water stratification breaks down during the winter, water from the deep mixes with the surface [102]. We suspect that the mixing of waters leads to a transfer of nutrients between water masses, causing an injection of phosphonates into the surface waters. Increased phosphonate substrate availability grants individuals capable of catabolizing phosphonates a boost to survival, facilitating population growth represented through increased gene relative abundance. Granted potential for phosphonate biosynthesis increases as well, though we can link this to the increased archaea population during winter months. Mixing waters as the basis of higher potential for phosphonate catabolism during the winter would explain why the sub-tropical waters displayed a greater seasonal affect than the sub-Antarctic as the warmer sub-tropical water would cause stronger stratification and more intense mixing [102] (Fig. 5) (Supplemental Table 9).

Considering this work, we question if enzyme cost plays a determinant role in the distribution and retention of phosphonate catabolizing genes in the ocean. Does trace metal availability affect the distribution of phosphonate catabolizing genes for a microbial community? The three enzymatic endpoints for 2-AEP catabolism discussed in this study: *phnA*, *phnX*, and *phnZ* involve different metals in the catalytic core: Zn, Mg, and Fe respectively. Availability of trace metals in the environment or other metal requirements within the cell may determine which 2-AEP catabolism strategy an individual retains. The large swathe of genes for the C-P lyase and multi-enzyme complex demands higher nutrient input for genome replication and protein construction than 2-AEP specific catabolism. However, is this difference measurably significant? And if so, are organisms with reduced energy limitations like aerobic anoxygenic photosynthetic bacteria able to dominate the niche in illuminated surface waters or are there other strategies for supporting the C-P lyase in surface and mesopelagic waters alike? Future work will continue to investigate some of these research questions to further the understanding of marine microbial phosphonate cycling and its contribution to P cycling.

## Supplementary information

Supplementary Dataset S1

Supplementary Materials

Supplementary Table S3

Supplementary Table S4

Supplementary Table S6

Supplementary Table S7

Supplementary Table S8

Supplementary Table S9

Supplementary Table S10

Supplementary Table S11

Supplementary Files S1

Supplementary Files S1

Supplementary Files S1

Supplementary Files S1

Supplementary Files S1

Supplementary Files S1

Supplementary Files S1

Supplementary Files S1

Supplementary Files S1

Supplementary Files S1

Supplementary Files S1

Supplementary Files S1

Supplementary Files S1

Supplementary Files S1

Supplementary Files S2

Supplementary Files S2

Supplementary Files S2

Supplementary Files S2

Supplementary Files S2

Supplementary Files S2

Supplementary Files S2

Supplementary Files S2

Supplementary Files S2

Supplementary Files S2

Supplementary Files S2

Supplementary Files S2

Supplementary Files S2

Supplementary Files S2

Supplementary Files S3

Supplementary Files S3

Supplementary Files S3

Supplementary Files S3

Supplementary Files S3

Supplementary Files S3

Supplementary Files S3

Supplementary Files S3

Supplementary Files S3

Supplementary Files S3

Supplementary Files S3

Supplementary Files S3

Supplementary Files S3

Supplementary Files S3

## Data Availability

MOTS sequence data is available within the NCBI-SRA archive under BioProject PRJNA605648: Munida Microbial Observatory Time-Series (MOTS) [HiSeq].
