# Supplementary material for: Global and seasonal variation of marine phosphonate metabolism
Source: ISME J. 2022 Jun 23;16(9):2198–212. doi: 10.1038/s41396-022-01266-z (PMC9381506; doi:10.1038/s41396-022-01266-z)
Supplement: Supplementary file 2 — Supplementary Materials [file 41396_2022_1266_MOESM2_ESM.pdf]

Supplementary Materials for

Title: "Global and seasonal variation of marine phosphonate metabolism"

Authors: Scott Lockwood[1,2], Chris Greening[3], Federico Baltar[2,4**], Sergio E. Morales[1*]

Correspondence to:
*E-mail: sergio.morales@otago.ac.nz
**E-mail: federico.baltar@univie.ac.at

This PDF file includes:
Supplementary Figures S1-S12
Supplementary Tables S1, S2, S5
Titles and legends for Supplementary Tables S3-S4, S6-S11

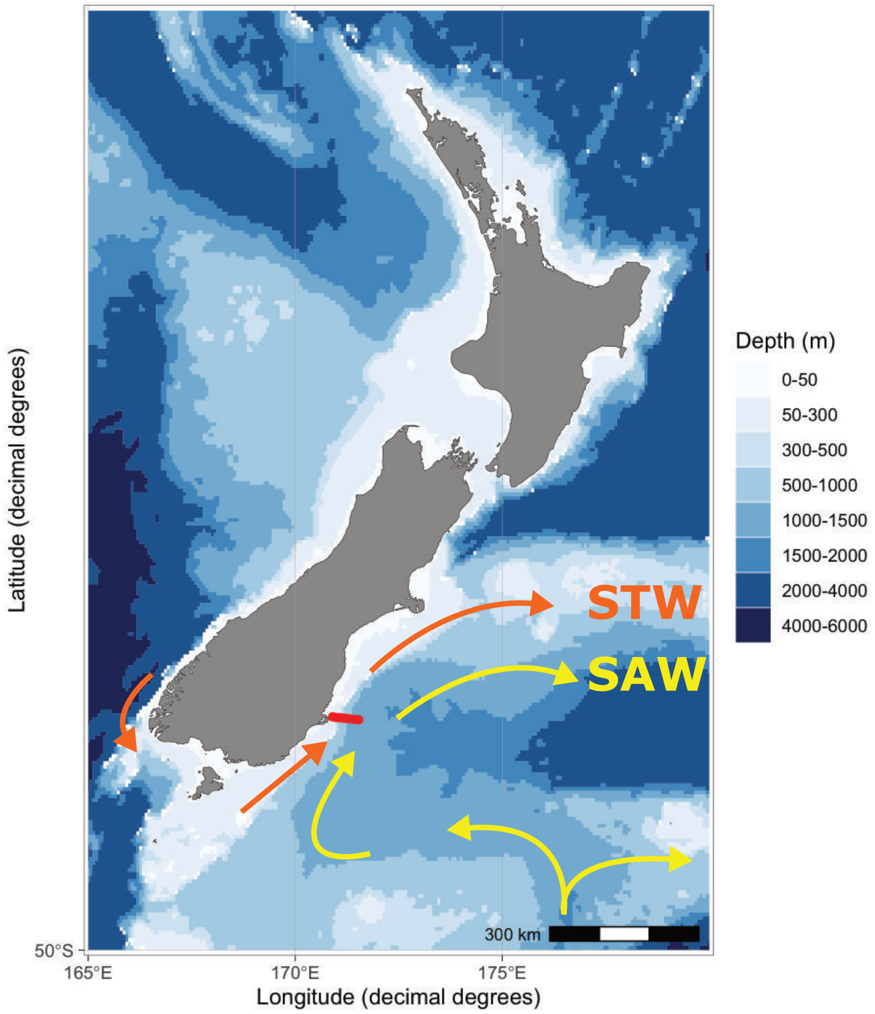

Supplementary Figure S1. Geography of the Munida Time-Series Transect (MOTS). The Munida Time-Series Transect is located 16 km offshore of Dunedin, New Zealand. The 65 km transect spanning eight sampling stations is marked by the red line. The Subtropical front (STW) and Sub-Antarctic front (SAW) are marked by the orange and green arrows, respectively.

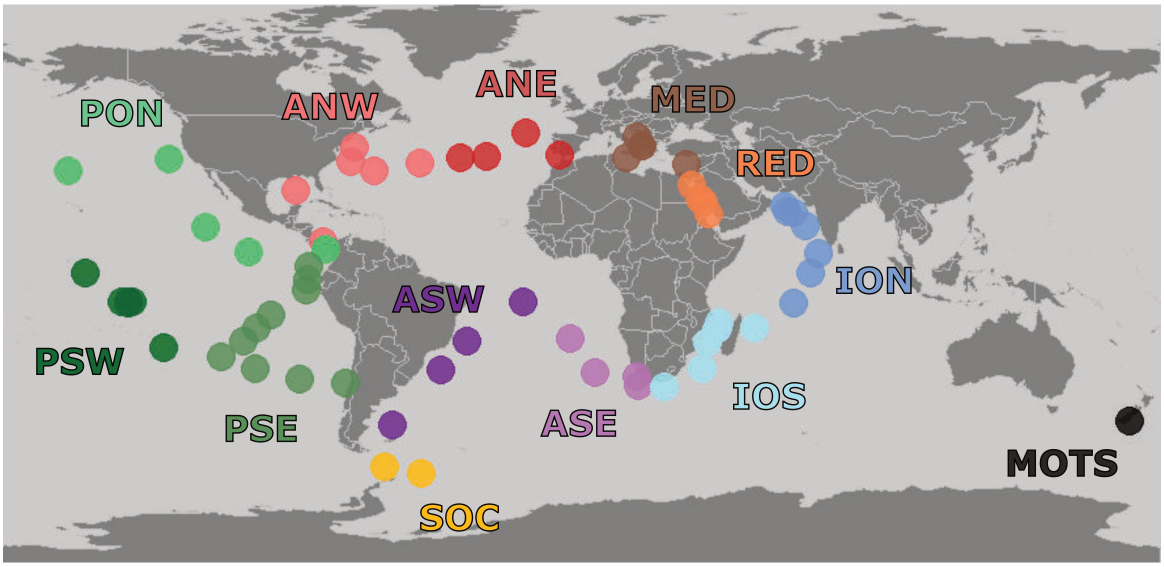

Supplementary Figure S2. Global sampling scheme for metagenomic analysis. Each circle represents a sampling site except for MOTS where the circle represents a transect. Sample selection and grouping (aside from MOTS) following “Nitrogen-fixing population of Planctomycetes and Proteobacteria are abundant in surface ocean metagenomes” Delmont *et al* 2018.

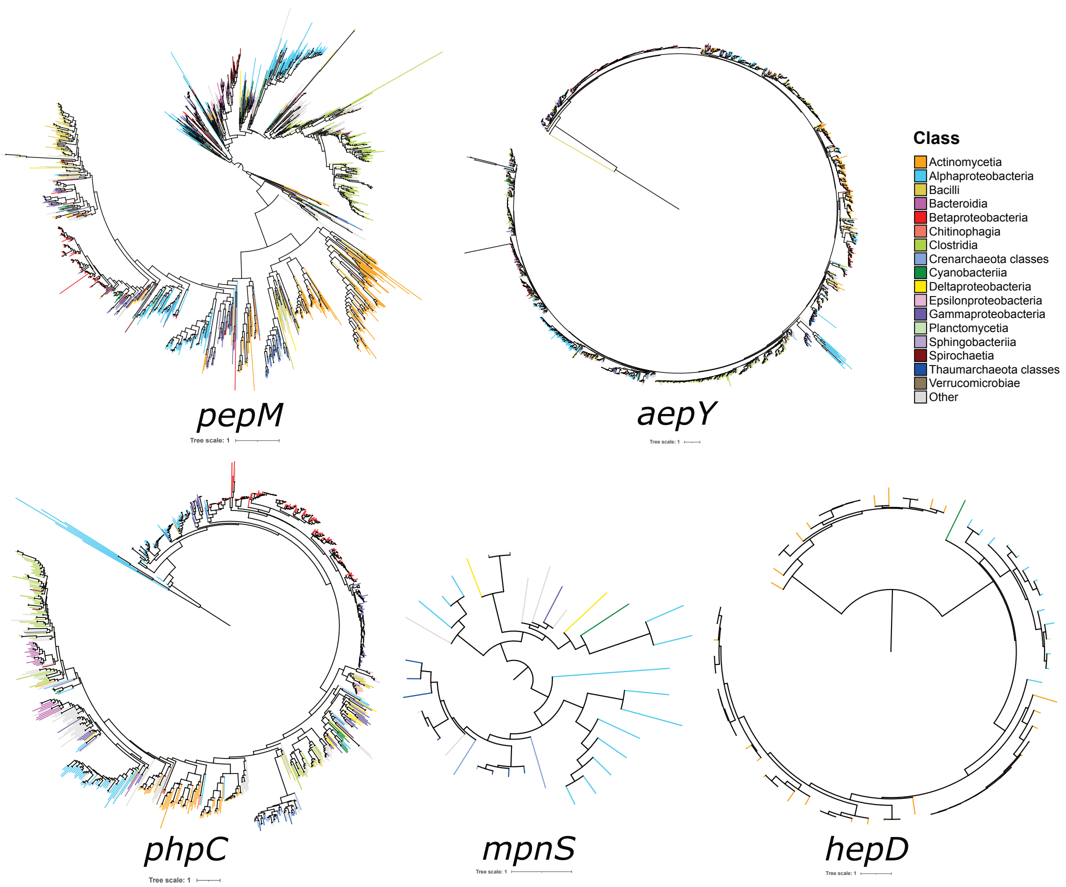

Supplementary Figure S3. Phosphonate production gene phylogenies.

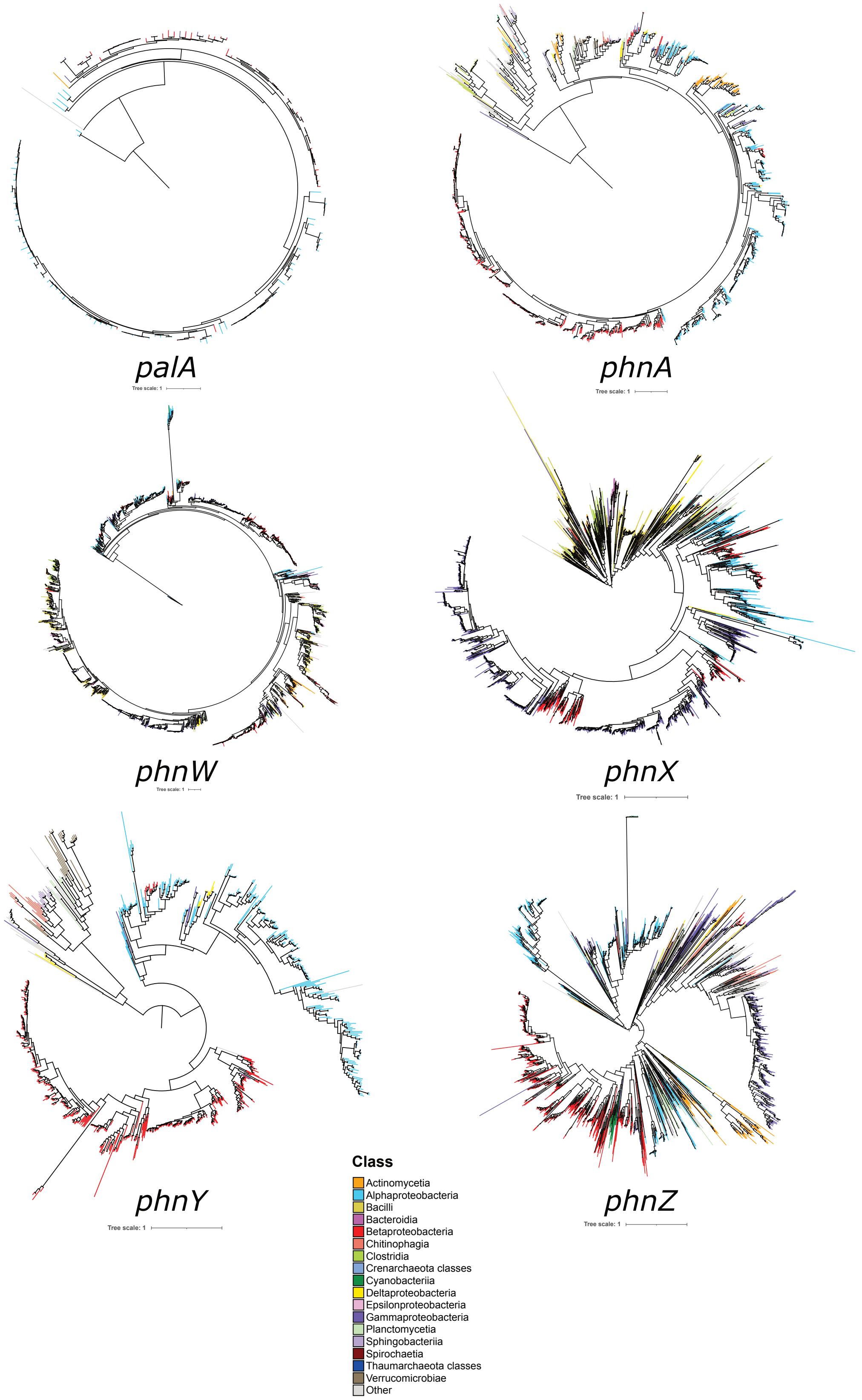

Supplementary Figure S4. Substrate-specific phosphonate catabolism gene phylogenies.

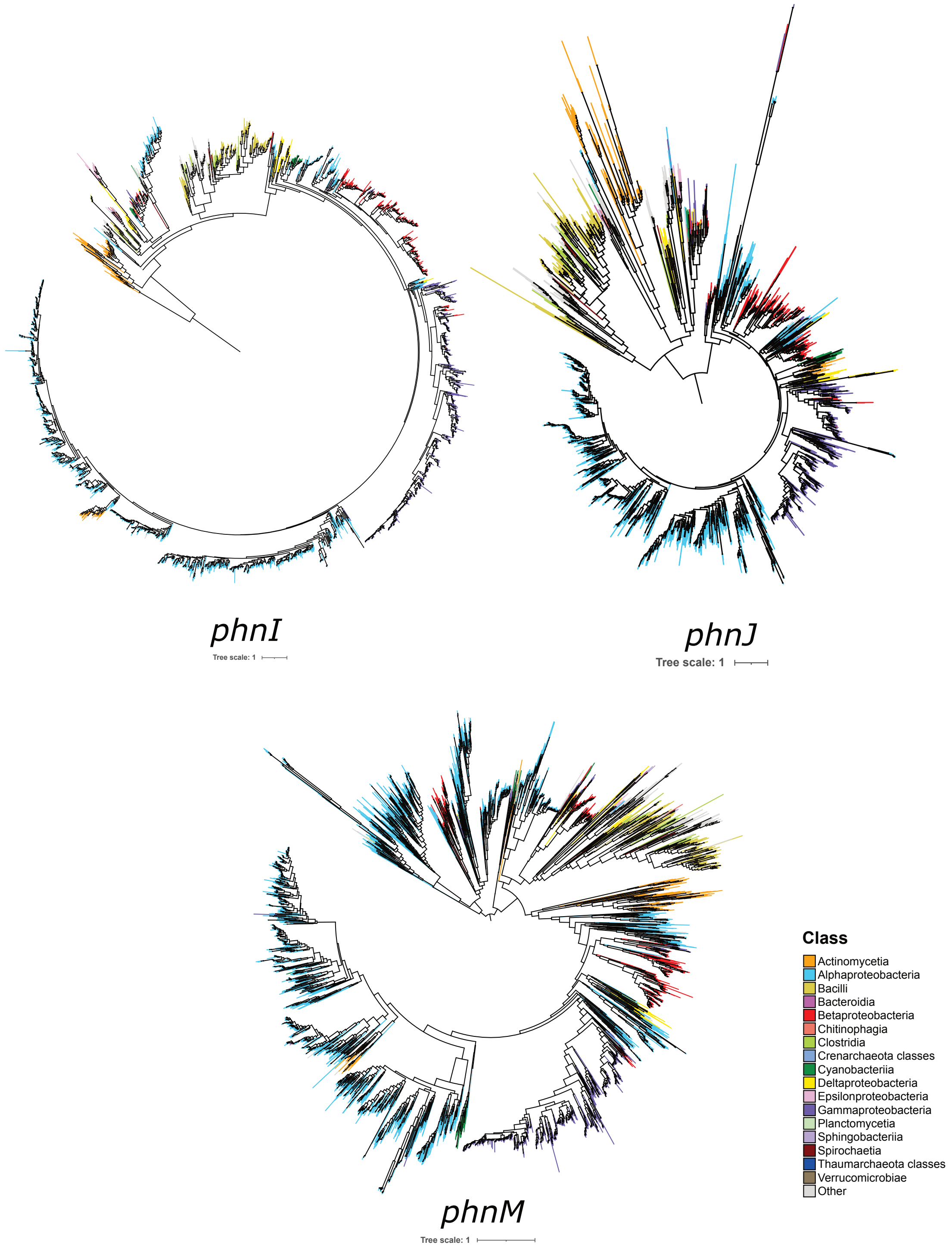

Supplementary Figure S5. Broad-specificity phosphonate catabolism (C-P lyase) gene phylogenies.

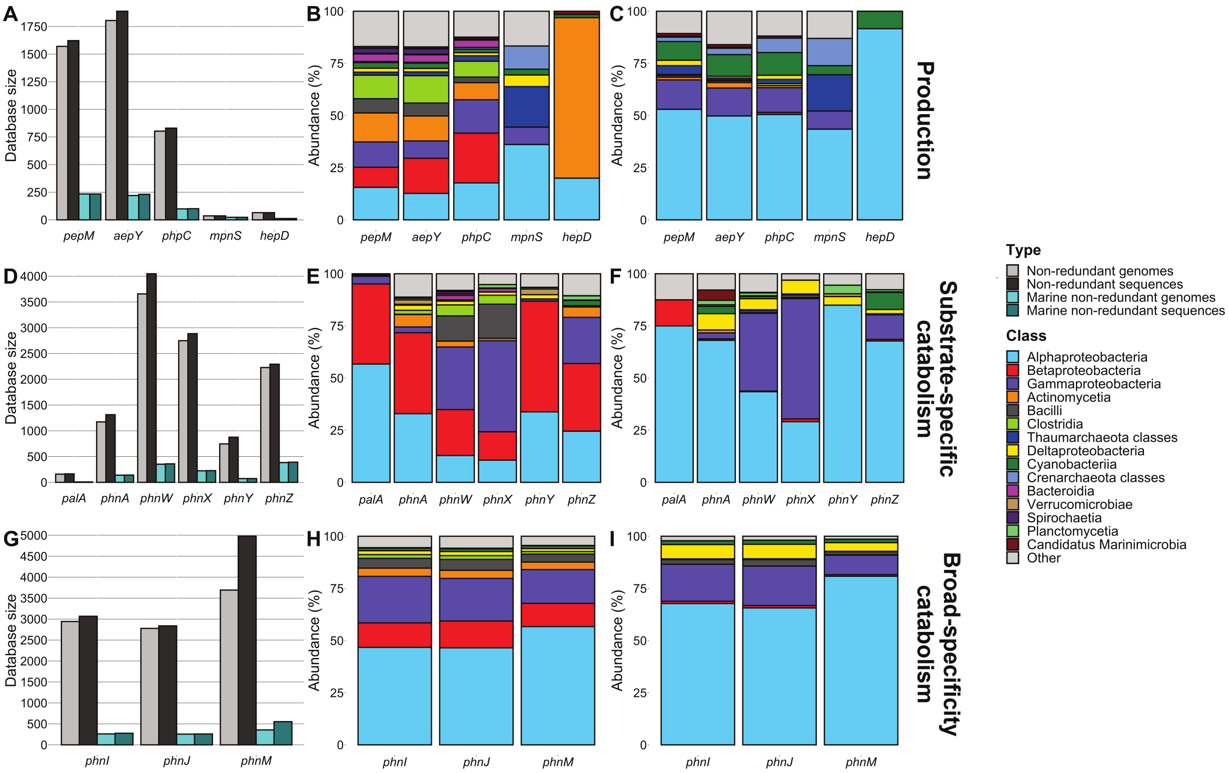

Supplementary Figure S6. Gene count and taxonomic composition of non-redundant (nr) databases. Number of sequences and genomes collected for study (A, D, G) with distribution of class-level taxa for all non-redundant sequences (B, E, H) and marine non-redundant (C, F, I) sequences. Results are shown for selected genes representing phosphonate (A-C) production, (D-F) substrate-specific catabolism, and (G-I) broad-specificity catabolism. The taxa shown are the 15 classes with the highest representation across all databases.

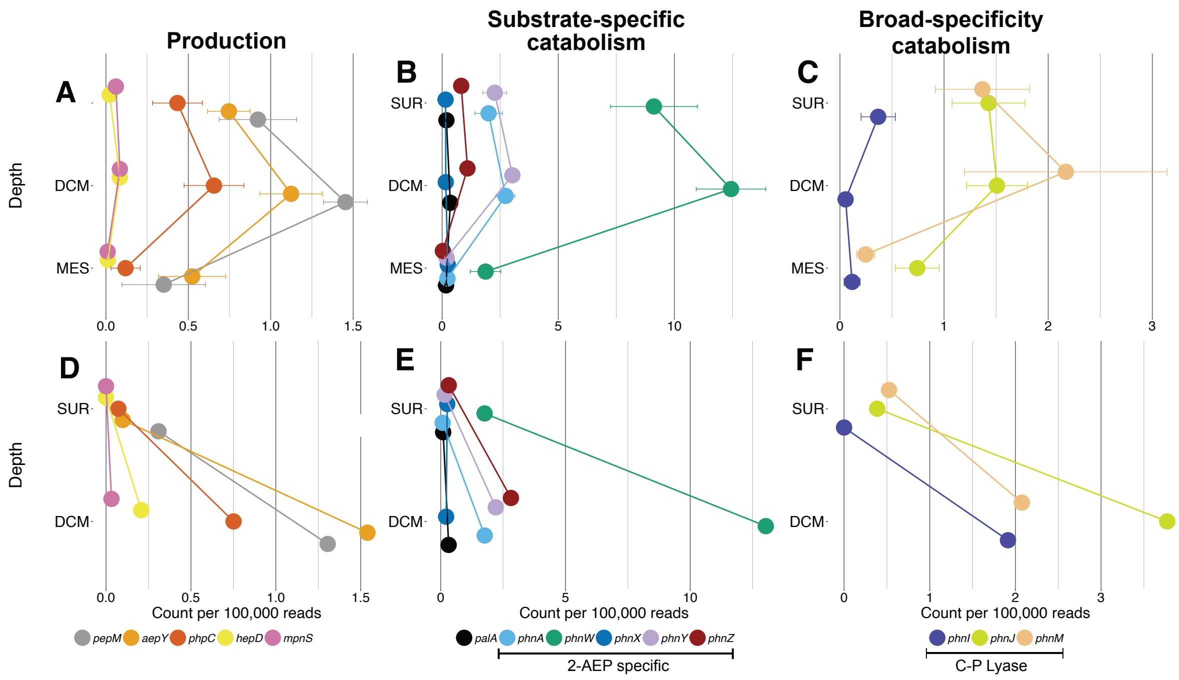

Supplementary Figure S7. Transcript counts of phosphonate cycling genes in global TARA oceans metatranscriptomes. Results of the TARA metatranscriptome DIAMOND search. Gene hits were normalized between samples by calculating the number of hits per 1000 reads. Results are shown for marker genes for A.) phosphonate production, B.) substrate-specific catabolism, and C.) broad-specificity catabolism. Relative abundance is shown separately for global TARA Oceans samples (top; covering ANE, ANW, ASE, ASW, ION, IOS, PON, PSE, PSW, RED, SOC samples) and Mediterranean Sea (bottom; comprising MED samples). Results are shown for surface (SUR), deep chlorophyll maximum (DCM), and, for the global samples, mesopelagic (MES) zones.

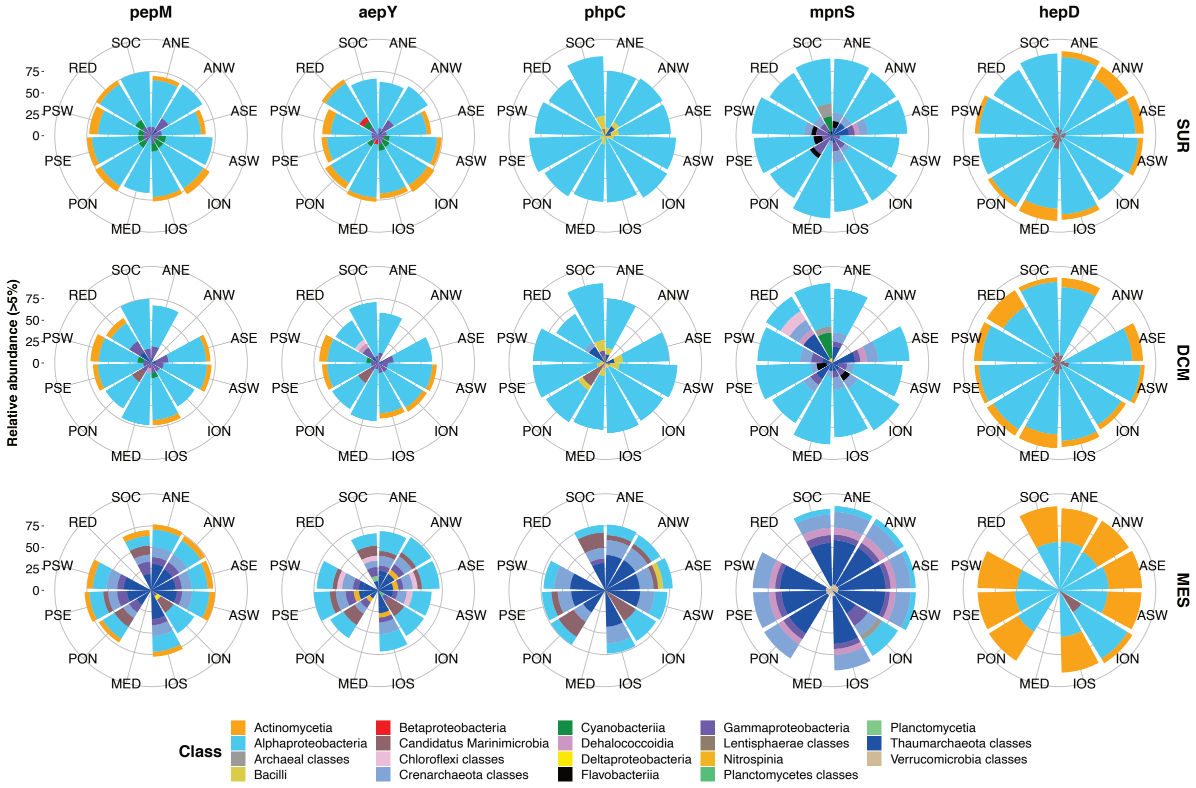

Supplementary Figure S8. Taxonomic distribution of phosphonate production genes across global ocean regions. Taxonomy for each gene are shown in three radar graphs in a column below the gene name. Each column shows the taxonomic spread for each gene at each sampling location and depth. Taxonomy displayed is at the class level and filtered so that a class must map to at least 5% of the gene hits for each location/depth. Genes that have few taxa shown with low total percentages indicates a diverse taxonomic range in the marine setting.

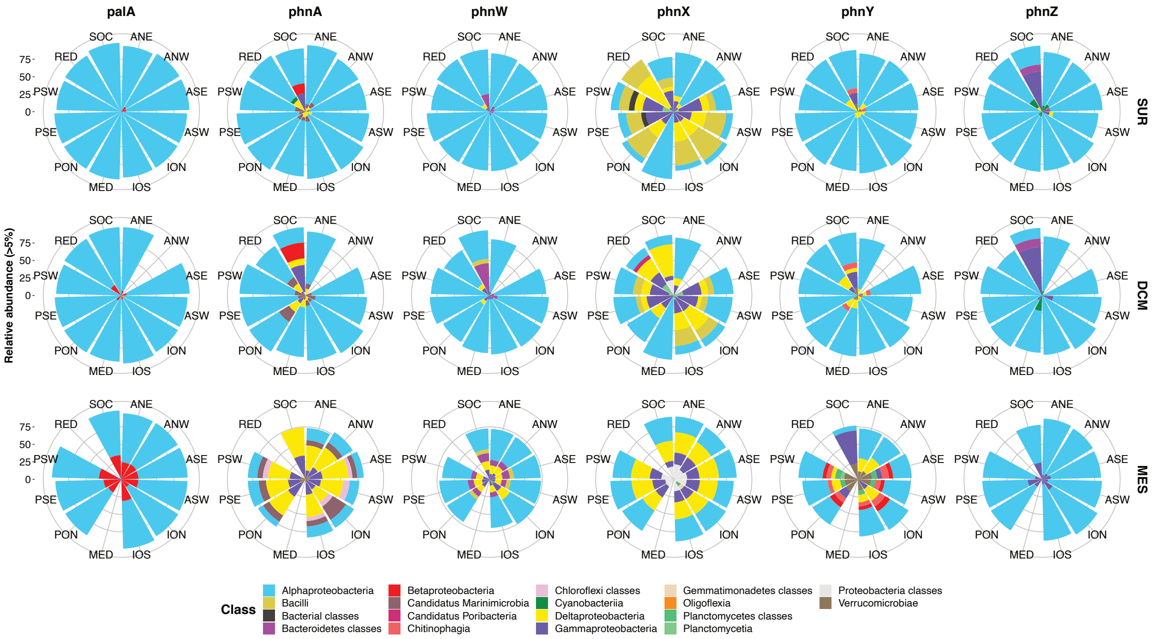

Supplementary Figure S9. Taxonomic distribution of substrate-specific phosphonate catabolism genes across global ocean regions. Taxonomy for each gene are shown in three radar graphs in a column below the gene name. Each column shows the taxonomic spread for each gene at each sampling location and depth. Taxonomy displayed is at the class level and filtered so that a class must map to at least 5% of the gene hits for each location/depth. Genes that have few taxa shown with low total percentages indicates a diverse taxonomic range in the marine setting.

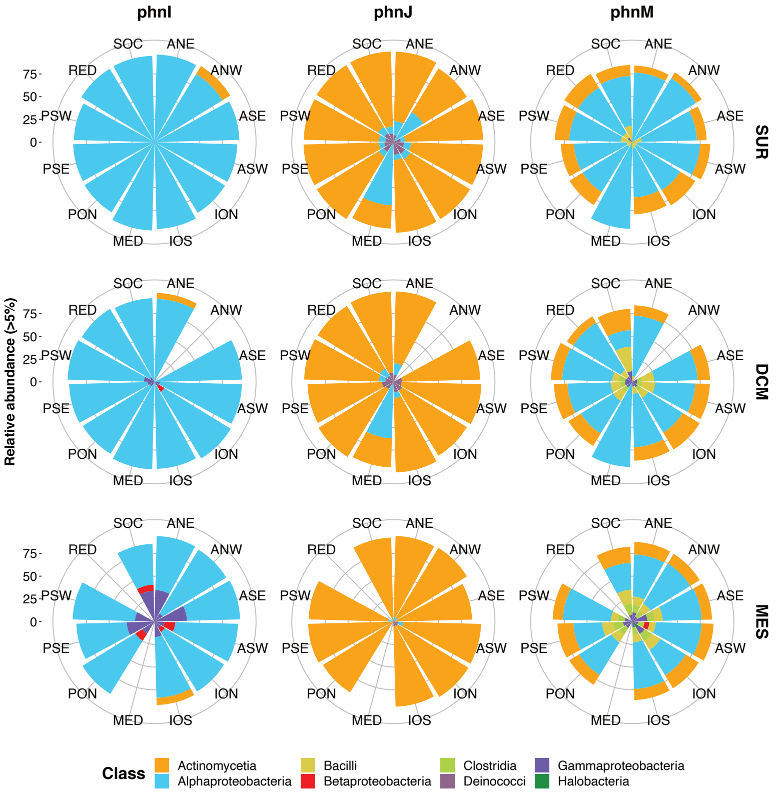

Supplementary Figure S10. Taxonomic distribution of broad-specificity (C-P lyase) phosphonate catabolism genes across global ocean regions. Taxonomy for each gene are shown in three radar graphs in a column below the gene name. Each column shows the taxonomic spread for each gene at each sampling location and depth. Taxonomy displayed is at the class level and filtered so that a class must map to at least 5% of the gene hits for each location/depth. Genes that have few taxa shown with low total percentages indicates a diverse taxonomic range in the marine setting.

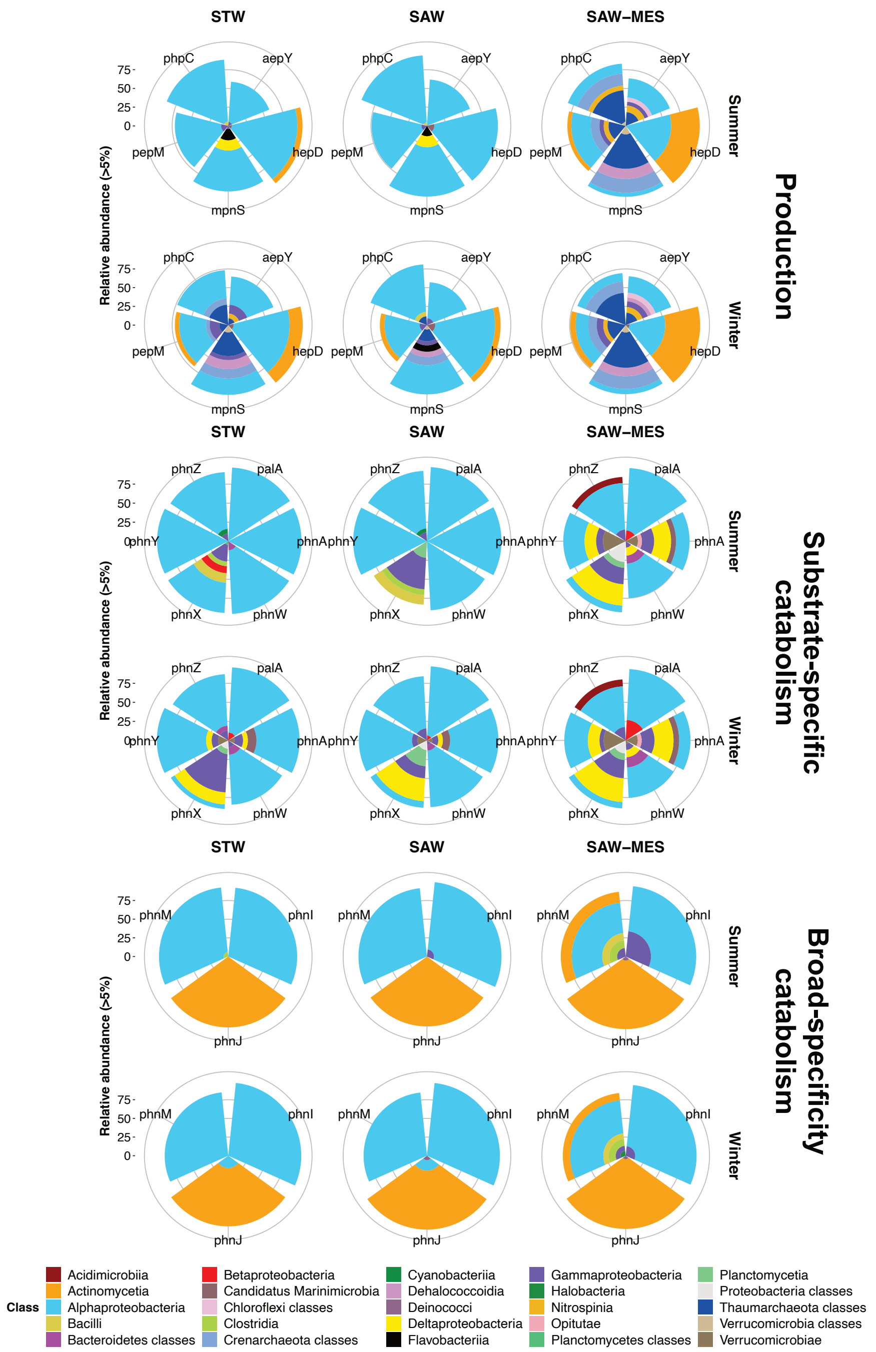

Supplementary Figure S11. Seasonal phosphonate cycling taxonomy in MOTS. Taxonomy at Class level for phosphonate A) production B) substrate-specific catabolism, and C) broad-specificity catabolism. Each gene's taxonomy was filtered so that any unique Class must map to at least 5% of the gene hits for each location/season.

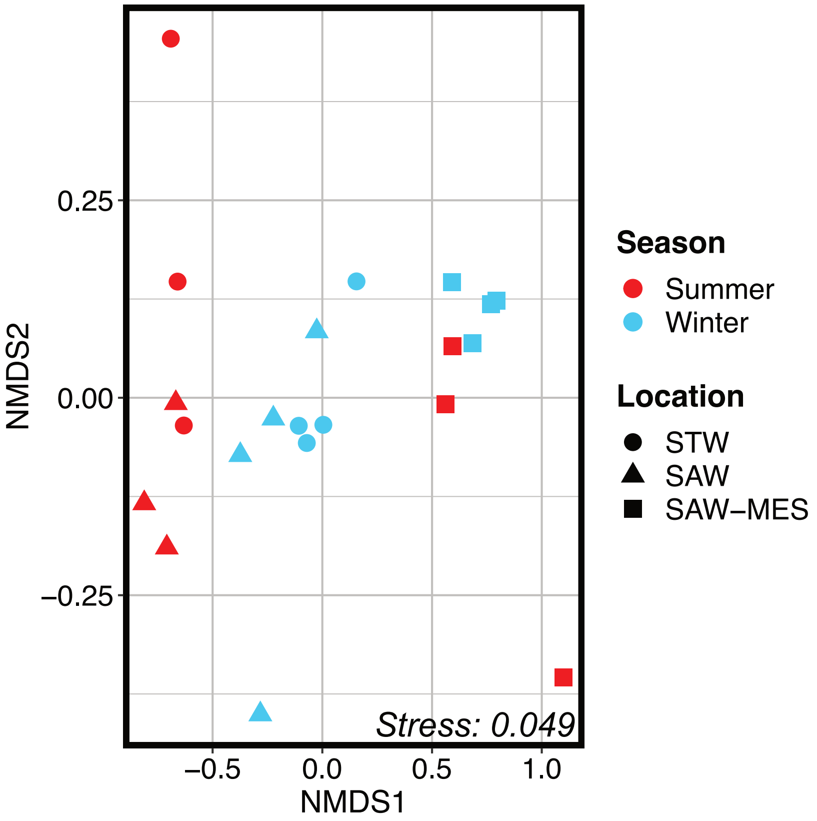

Supplementary Figure S12. Hierarchical clustering of deterministic factors influencing abundance and taxonomy of phosphonate cycling genes in the Munida Time Series Transect (MOTS). NMDS using Bray-Curtis dissimilarity of pooled relative abundance and taxonomic data for each water mass and time point. Shape designates water mass: STW (square), SAW (circle), and SAW-MES (triangle). Color represents season: summer (red) and winter (blue).

Supplementary Table S1. Phosphonate cycling gene database annotations. List of annotations (KEGG, TIGRfam, pfam, or COG) used to search for genes in JGI/IMG and GORG-Tropics databases.

Gene	Annotation
pepM	KO:K01841
pepM	TIGR02320
pepM	TIGR02321
aepY	KO:K09459
aepY	TIGR03297
phpC	KO:K12904
phpC	KO:K12901
phpC	TIGR03405
mpnS	KO:K18049
hepD	KO:K12905
hepD	KO:K18049
palA	KO:K19669
palA	TIGR02321
phnA	KO:K19670
phnA	TIGR02335
phnW	KO:K03430
phnW	TIGR02326
phnW	TIGR03301
phnX	KO:K05306
phnX	TIGR01422
phnY	KO:K19266
phnY	TIGR03250
phnZ	TIGR00277
phnZ	COG4341
phnI	KO:K06164
phnJ	KO:K06163
phnM	KO:K06162
phnM	TIGR02318

Supplementary Table S2. Phosphonate cycling gene metal requirements and conserved residues. Conserved residues (CR) used for curation of databases.

Gene	Metal	CR1	CR2	CR3	CR4	CR5	CR6	CR7
pepM	Mg 2+ per subunit	E114	D115	K116	N122	S123	x	x
aepY	Mg 2+, Ca 2+, or Mn 2+	G233	G259	D260	G261	x	x	x
phpC	Fe cation	D199	H268	H282	x	x	x	x
mpnS	Fe 2+	H148	Q152	F162	I/V184	H190	x	x
hepD	Fe 2+ per subunit (homodimer)	H148	A/Q152	I/Y162	E/G184	H190	x	x
palA	Co 2+, Mg 2+, or Mn 2+	E110	D111	K112	T118	S119	x	x
phnA	(2x) Zn 2+	D211	H215	D250	H251	H377	x	x
phnW	Mg 2+	D168	K194	R340	x	x	x	x
phnX	Mg 2+	G47	H53	x	x	x	x	x
phnY	--------------------	R108	H159	R447	x	x	x	x
phnZ	(2x) Fe 2+	H58	D59	H62	H104	K108	R158	D161
phnI	--------------------	H328	H333	x	x	x	x	x
phnJ	[4Fe-4S] per subunit	C241	C244	C266	C272	x	x	x
phnM	--------------------	H59	D61	H230	x	x	x	x

Supplementary Table S5. Gene database diversity statistics. Results from Shannon index and evenness calculation based on the taxonomic distribution within each database (nr and redundant; all and marine). Calculated using the *vegan* package in R.

Gene	All/Marine?	Redundant?	n	Shannon index (H)	Evenness
pepM	All	redundant	2396	2.662946	0.5947611
aepY	All	redundant	2290	2.759192	0.6101992
phpC	All	redundant	937	2.490944	0.603553
mpnS	All	redundant	37	1.990226	0.8299886
hepD	All	redundant	71	0.618116	0.4458764
palA	All	redundant	207	0.8578151	0.5329905
phnA	All	redundant	1875	1.668112	0.4491933
phnW	All	redundant	5388	2.13288	0.5003613
phnX	All	redundant	3687	1.799786	0.4674589
phnY	All	redundant	1479	1.084125	0.3618898
phnZ	All	redundant	2800	1.798004	0.5142286
phnI	All	redundant	4340	1.62901	0.4331092
phnJ	All	redundant	4255	1.639937	0.4360142
phnM	All	redundant	6269	1.498769	0.3937226
pepM	All	nr	1622	2.92685	0.6553763
aepY	All	nr	1888	2.932488	0.6500953
phpC	All	nr	830	2.5979	0.6294682
mpnS	All	nr	36	1.956316	0.815847
hepD	All	nr	65	0.6521489	0.470426
palA	All	nr	164	0.8724286	0.5420704
phnA	All	nr	1313	1.91404	0.5154174
phnW	All	nr	4048	2.254338	0.5342655
phnX	All	nr	2754	1.887298	0.4929419
phnY	All	nr	876	1.280014	0.4272792
phnZ	All	nr	2292	1.883331	0.5386321
phnI	All	nr	3067	1.744519	0.4667399
phnJ	All	nr	2838	1.779082	0.4730092
phnM	All	nr	4976	1.55154	0.4075855
pepM	Marine	redundant	256	1.756179	0.5270323
aepY	Marine	redundant	243	1.921115	0.5765296
phpC	Marine	redundant	104	1.611334	0.6105718
mpnS	Marine	redundant	24	1.734743	0.8342352
hepD	Marine	redundant	12	0.286836	0.4138168
palA	Marine	redundant	8	0.7356219	0.6695919
phnA	Marine	redundant	224	1.008918	0.3638904
phnW	Marine	redundant	446	1.534501	0.4557073
phnX	Marine	redundant	326	1.189178	0.4506071
phnY	Marine	redundant	162	0.4102825	0.2108435
phnZ	Marine	redundant	538	1.121457	0.40448
phnI	Marine	redundant	435	0.8582492	0.3579177

phnJ	Marine	redundant	423	0.8751531	0.3649672
phnM	Marine	redundant	725	0.6951196	0.2710071
pepM	Marine	nr	234	1.848505	0.5673573
aepY	Marine	nr	231	1.826452	0.5605886
phpC	Marine	nr	101	1.595903	0.6047246
mpnS	Marine	nr	23	1.689705	0.8125762
hepD	Marine	nr	12	0.286836	0.4138168
palA	Marine	nr	8	0.7356219	0.6695919
phnA	Marine	nr	141	1.384798	0.4994605
phnW	Marine	nr	359	1.56859	0.4814438
phnX	Marine	nr	218	1.170869	0.4711925
phnY	Marine	nr	73	0.6497767	0.3626473
phnZ	Marine	nr	391	1.248114	0.4501621
phnI	Marine	nr	276	1.090509	0.4547774
phnJ	Marine	nr	259	1.135297	0.4734557
phnM	Marine	nr	553	0.7962575	0.3104379

Titles and legends for additional supplementary tables:

Supplementary Table S3. TARA sample metadata. Environmental metadata for all TARA samples used in this study.

Supplementary Table S4. MOTS sample metadata. Environmental metadata for all MOTS samples used in this study.

Supplementary Table S6. Change in phosphonate cycling gene relative abundance between depths or locations in global oceans. ANOVA of mean gene relative abundance between consecutive depths from the TARA Oceans dataset and between the global and Mediterranean (MED) samples. MED samples are subset from the other 11 locations (Global) to a separate analysis due to a nutrient environment that poorly represents the global oceans.

Supplementary Table S7. Specific comparisons between depths for changes in phosphonate cycling gene relative abundance in global oceans. Results from Tukey HSD post-hoc test following up the ANOVA analysis displayed in Supplementary Table S6.

Supplementary Table S8. Breakdown of specific taxonomic contributions to overall relative gene abundance by sample for TARA analysis. A breakdown for each phosphonate cycling gene showing the contribution of a specific taxonomy to the gene's relative abundance in the TARA dataset. Information shown was aggregated by taxonomy, not reference, so some rows may represent more than one sequence from the databases.

Supplementary Table S9. Changes in phosphonate cycling gene relative abundance between locations or seasons in MOTS. ANOVA of mean gene relative abundance between depths or seasons for phosphonate cycling genes detected in the MOTS dataset.

Supplementary Table S10. Breakdown of specific taxonomic contributions to overall relative gene abundance by sample for MOTS analysis. A breakdown for each phosphonate cycling gene showing the contribution of a specific taxonomy to the gene's relative abundance in the MOTS dataset. Information shown was aggregated by taxonomy, not reference, so some rows may represent more than one sequence from the database.

Supplementary Table S11. Phosphate regression statistics.
